# Distinct mammary stem cells orchestrate long-term homeostasis of adult mammary gland

**DOI:** 10.1038/s41421-025-00794-0

**Published:** 2025-04-15

**Authors:** Zuobao Lin, Yajing Guo, Huiru Bai, Xiaoqin Liu, Meizhen Lin, Yue Zhang, Ruolan Tang, Tian’en Hu, Lili Yu, Chunhui Wang, Shang Cai

**Affiliations:** 1 School of Life Sciences, Fudan University, Shanghai, China; 2 Westlake Laboratory of Life Sciences and Biomedicine, School of Life Sciences, Westlake University, Hangzhou, Zhejiang China; 3 Westlake Disease Modeling lab, Westlake Laboratory of Life Sciences and Biomedicine, Hangzhou, Zhejiang China; 4 Key Laboratory of Growth Regulation and Translational Research of Zhejiang Province, School of Life Sciences, Westlake University, Hangzhou, Zhejiang China; 5 Institute of Biology, Westlake Institute for Advanced Study, Hangzhou, Zhejiang China; 6 College of Life Sciences, Zhejiang University, Hangzhou, Zhejiang China

**Keywords:** Multipotent stem cells, Mammary stem cells

## Abstract

The murine mammary gland is sustained by distinct pools of stem cells that are limited in space and time, exhibiting both unipotency and bipotency. However, the specific identities of the bipotent and unipotent mammary stem cells remain unclear. In this study, we investigated spatial heterogeneity of the mammary gland at the single-cell transcriptional level. We found that mammary basal cells exhibited spatially distinct populations and characteristics, which can be further divided based on the expression of CD34 and CD200 markers. Notably, CD34^−^CD200^+^ basal cells enriched at the nipple region demonstrated strong long-term self-renewal ability and possessed the highest stem cell frequency, while CD34^+^CD200^−^ basal cells enriched in the terminal end buds (TEBs) showed reduced stem cell potency. Through lineage tracing experiments based on their signature genes, we discovered that Bcl11b^+^ cells were enriched in the CD34^−^CD200^+^ population and exhibited bipotency even in the postnatal mammary gland, with an increasing contribution to mammary epithelia observed during long-term tracing and after multiple rounds of pregnancies. Conversely, lineage tracing of Sema3a^+^ cells, enriched in the CD34^+^CD200^−^ population, predominantly revealed their unipotent nature and significant contribution during alveologenesis. Notably, the Bcl11b^+^ cells displayed a slow response to pregnancy but contributed to long-term mammary homeostasis, in contrast to the rapid response observed in Sema3a^+^ cells. In addition, Bcl11b progenies survived much better than Sema3a progenies during involution stage, thereby exhibiting increased coverage in the mammary gland after multiple rounds of pregnancies. Importantly, depletion of Bcl11b in Krt14^+^ mammary basal cells resulted in reduced bipotency of mammary stem cells and impaired their long-term contribution to the mammary gland. Overall, our study identifies distinct bipotent and unipotent populations of mammary basal cells with different dynamic properties that play critical roles in maintaining postnatal mammary homeostasis. These findings are crucial for advancing our understanding of breast health and breast cancer research.

## Introduction

The mammary gland is a highly specialized organ that develops postnatally and undergoes significant morphological changes during reproduction cycles^1–4^. Its dynamic architecture and the functional integrity are maintained by multiple lineages of cells that are organized in a hierarchical order with distinct long-lived stem cells at the top^5,6^. Understanding the hierarchical orchestration of the mammary homeostasis is instrumental for comprehending the normal mammary physiology as well as cancer transformation^5–7^.

Through the mammary transplantation and lineage tracing assays, multipotent mammary stem cell populations have been identified under various definitions with molecular markers including CD49f^+^CD24^med^/CD29^+^CD24^+^, Axin2^+^^8,9^, Procr^+^^10^, Lgr5^+^^11–13^ and Dll1^+^^5,14,15^. In addition, mammary stem cells can reside in quiescent state with highly expressed transcription factor Bcl11b^16^, which suppresses cell cycle activity and prevents stem cell exhaustion, or with cell surface marker CD1d^17^ and Tspan8^11^. However, lineage tracing analysis in recent years revealed that the existence of bipotent mammary stem cells in adult mammary gland is of question^11,18–21^. Specifically, lineage tracing using *Krt14-CreERT2*^18^, *Axin2-CreERT2*^8^, *Notch-CreERT2*^22^ and sporadic tracing assays^23^ suggest that under physiological condition, bipotent mammary stem cells mainly exist during embryonic stage, while unipotent stem cells are predominant in the postnatal mammary gland^8,18^. The transition from bipotent to unipotent mammary stem cells is thought to be driven by TNFα expression in luminal cells^24,25^. Consistent with this idea, long-lived luminal unipotent progenitors have been identified for both hormone receptor positive and negative luminal cells^26,27^. However, other tracing assays with Krt5^28^, Procr^10^ and Dll1^14^ have shown evidence of bipotent stem cells in the adult mammary gland. It remains highly controversial how adult mammary gland is maintained by what types of stem cells, and the identities of the long-lived bipotent mammary stem cells and unipotent mammary stem cells in the mammary gland are poorly understood. It is also unresolved whether the various cellular states of the same stem cell population, or the distinct stem cell populations resulted in the variable tracing outcomes under physiological condition. Therefore, it is imperative to comprehensively investigate the heterogeneity of basal cells and their contributions to mammary homeostasis.

In this study, we characterized the spatial distribution of mammary cells at the single cell level and found that mammary basal cells can be categorized into three distinct populations based on the expression of CD34 and CD200. The CD34^−^CD200^+^ quiescent cells exhibited the highest efficiency in mammary reconstitution. Lineage tracing assays demonstrated that both bipotent and unipotent mammary stem cells co-existed in postnatal mammary gland. The bipotent cells exhibited a slow but long-lasting contribution, while the unipotent cells displayed a fast but limited contribution. Loss of long lived bipotent mammary stem cells in adult mouse impairs architectural and functional integrity of the mammary gland. Therefore, our findings suggest that both bipotent and unipotent mammary stem cells play crucial roles in maintaining normal mammary gland physiology.

## Results

### Mammary basal cells are spatially heterogeneous at the transcriptome level

**Fig. 1:**
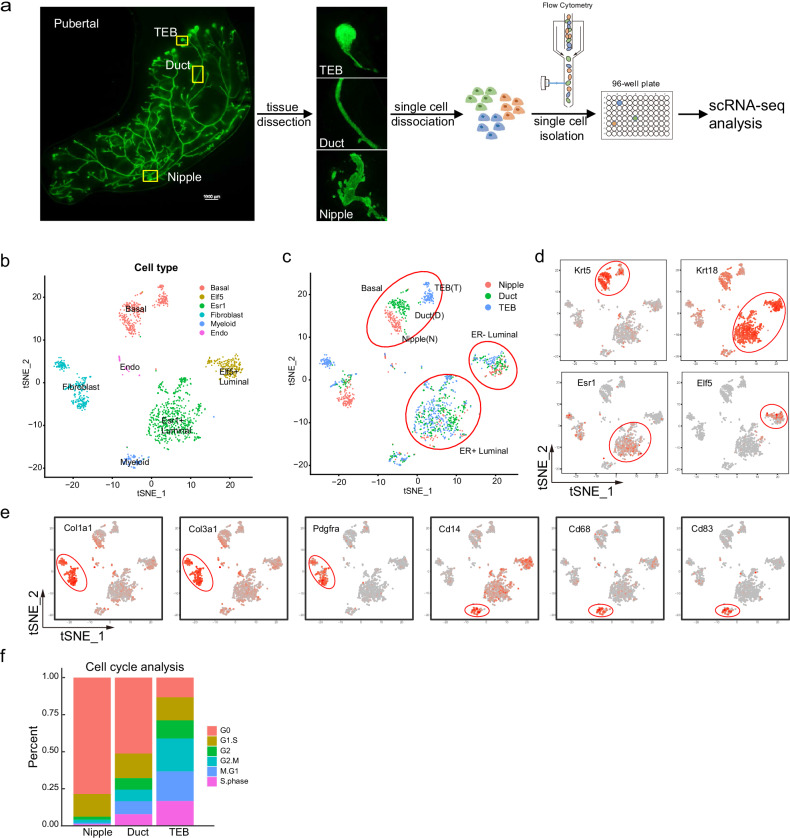
scRNA-seq analysis reveals the spatial heterogeneity of pubertal mammary gland. **a** Schematic workflow for scRNA-Seq in **b**−**f**. Various mammary tissue structures, including TEBs, ducts and nipples, from mammary glands of 5-week-old *K14-Cre/R26-mTmG* mice, were processed into single-cell suspensions and subjected to 3’ UTR single-cell SMART-seq. Scale bar, 1000 μm. **b**, **c** t-SNE plots show the clustering of mammary cells from various locations. Distinct mammary populations are displayed in different colors in **b**. The anatomical locations of cells are shown in different colors in **c**, indicating that mammary epithelial cells from different locations cluster well into basal, ER^−^ luminal, and ER^+^ luminal populations (in red circles), while still exhibiting heterogeneity within basal and stromal cells based on spatial distribution. **d** Signature gene expression in mammary epithelial cells. *Krt5* for basal cells, *Krt18* for luminal cells, *Esr1* for ER^+^ luminal cells, and *Elf5* for ER^−^ luminal cells. **e** Signature gene expression in fibroblasts (*Col1a1*, *Col3a1* and *Pdgfra*) and myeloid cells (*Cd14*, *Cd68* and *Cd83*). **f** Proportion of cells in different cell cycle phases for basal cells in TEB, duct and nipple.

Previous studies have provided single-cell resolution profiling of the murine mammary gland transcriptome^29–33^, however the spatial heterogeneity exhibited by mammary stem cells has not been addressed enough in these studies. Recognizing the importance of understanding the molecular identity of mammary cells in different locations, we surgically dissected single mammary fragments from six terminal end buds (TEBs), six ducts, and four nipples, and processed each individual structure into single cells followed by single-cell RNA sequencing (scRNA-seq) to characterize their molecular profiles (Fig. 1a). After stringent quality control (Supplementary Fig. S1a, b), we recovered 3198 cells from individual TEBs, ducts and nipples. t-distributed stochastic neighbor embedding (t-SNE) plot showed that mammary cells from different locations clustered mainly into six populations: basal, ER^+^ luminal, ER^−^ luminal, fibroblasts, endothelia and myeloid cells (Fig. 1b, c), with their corresponding signature genes expressed respectively (Fig. 1d, e). When we focused on the mammary epithelial cells, we found that luminal cells from different locations intermingled well with each other, indicating similar transcriptome. In contrast, the basal cells were subclustered into three distinct populations that are clearly associated with their locations (Fig. 1c; Supplementary Fig. S1c) and the location specific signature genes including TEB (*Mycn*, *Csn3*, *Slc6a15*, *Tbx2*), Duct (*Actg2*, *Alpl*, *Cyp1b1*, *Ereg*, *Igfbp2*, *Ttc9*), and Nipple (*Tspan8*, *Lgr5*, *Sfrp1*, *Ccdc129*, *Slpi*) (Supplementary Figs. S2a, b, S3a, b). This suggests that the spatial heterogeneity of murine mammary epithelia is mainly manifested by basal cells.

To further dissect the molecular identity of the three basal populations, we performed pathway enrichment analysis. Nipple basal cells showed higher activity in survival-associated pathways such as PI3K-Akt, and cytoskeleton associated pathways such as focal adhesion, regulation of actin cytoskeleton, ECM-receptor interaction, and Rap1 signaling pathway (Supplementary Fig. S1d). In contrast, TEB basal cells clearly showed activity-associated pathways including lipid metabolism, cell cycle and DNA replication (Supplementary Fig. S1d). We also assessed the expression of several stem cell signature genes (Supplementary Fig. S2a–c). Among them, *Dll1* and *Trp63* exhibited broad distribution across all basal populations, whereas *Tspan8*, *Lgr5* and *Procr* showed a more restricted distribution. Specifically, *Tspan8*, a previously reported spatially restricted stem cell marker^11^, was highly enriched in nipple basal cell population (Supplementary Fig. S2a–c). Bcl11b, reported in our previous work^16^, is an important regulator for mammary epithelial stem cell quiescence. Although its RNA expression was barely detectable in this scRNA-seq analysis (data not shown), probably due to the low expression of transcription factors or insufficient sequencing depth, its activity manifested by its targets^34^ was enriched in the nipple population (Supplementary Fig. S3c). The activity of Bcl11b regulon based on single cell analysis suggested the same trend (Supplementary Fig. S3d). Further qPCR quantification and immunofluorescence staining analysis confirmed Bcl11b’s expression gradient (Supplementary Fig. S3e–g). Consistent with these features, cell cycle analysis of different basal populations showed that nipple basal cells were predominantly composed of G0 quiescent cells^11^ while most of TEB cells were in the cell cycle (Fig. 1f).

To further analyze the cell fates associated with location, we extracted the basal cells and performed trajectory analysis using the SCORPIUS R package11 (Supplementary Fig. S3h, i). The result showed that cells from the nipple region were enriched at earlier stage on the pseudotime, followed by duct cells, and then the TEB. We also conducted differential gene expression analysis along the pseudotime trajectory and defined three gene sets corresponding to the nipple, duct, and TEB (Supplementary Fig. S3i). Canonical stem cell marker genes such as *Sox9*, *Snai2*, and *Id4* exhibited higher activity in the nipple and the lowest activity in the TEB (Supplementary Fig. S3i). Genes regulated by *Bcl11b* also showed the highest activity in the nipple (Supplementary Fig. S3i). Additionally, we used two other trajectory inference methods: TSCAN (version 1.34.0, 2022) and Slingshot ot12. The pseudotime inferred by these three methods was significantly correlated (Supplementary Fig. S3j–l), suggesting a robust cell fate dynamics associated with locations.

Taken together, these findings suggest that basal cells are spatially heterogeneous with molecular features (Supplementary Figs. S2a, b, 3a–g, i) associated with distinct cell states/fates. This spatial feature provides valuable insights into the molecular identity and function of the postulated inactive stem cells and proliferative basal progenitor cells.

### Mammary basal cells contain functionally distinct populations

**Fig. 2:**
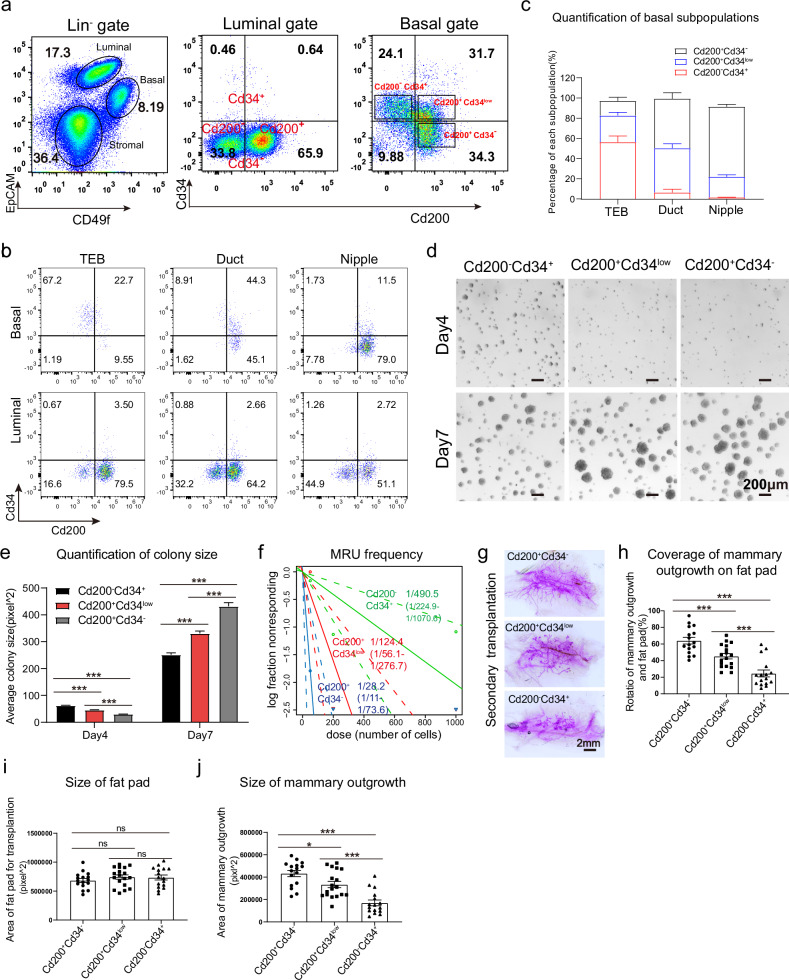
CD200 and CD34 identify three distinct basal subpopulations. **a** FACS plots showing three distinct basal subpopulations characterized by surface markers CD200 and CD34 (data were collected from BD FACSAria Fusion. The negative gate of CD34 and CD200 was set according to the luminal cells). **b** Representative FACS plots showing the expression of CD200 and CD34 in basal or luminal cells of TEB, duct and nipple (data collected from CytoFLEX). **c** Statistical analysis of percentages of different basal subpopulations categorized by CD200 and CD34 FACS staining in TEB, duct, or nipple as shown in **b**. Statistical analysis was performed using two-tailed unpaired *t*-test. Data were presented as means ± SEM, *n* = 3. **d** Colony formation assay for CD200^−^CD34^+^, CD200^+^CD34^low^, CD200^+^CD34^−^ basal subpopulations. Representative outgrowth images of 3 repeated wells on day 4 and day 7 for each subpopulation are shown. Scale bars, 200 μm. **e** Colony sizes in **d** were measured and plotted in the bar graph. Statistical analysis was performed using two-tailed unpaired *t*-test. Data were presented as means ± SEM, *n* = 3, ***P < 0.001. **f** Extreme limiting dilution analysis (ELDA) plot of the repopulating ability of FACS-sorted CD200^−^CD34^+^, CD200^+^CD34^low^ or CD200^+^CD34^−^ basal subpopulations under transplantation. *n* = 16−18. **g** Representative images of reconstituted mammary gland derived from the secondary transplants of CD200^−^CD34^+^, CD200^+^CD34^low^ or CD200^+^CD34^−^ basal subpopulations. Scale bar, 2 mm, *n* = 16−18. **h**–**j** Statistical analysis of the secondary transplant in **g**. The relative ratio of area covered by reconstituted mammary gland in the de-epithelialized fat pads are shown in **h**, and the absolute size of recipient fat pads and mammary outgrowths are shown respectively in **i**, **j**. Statistical analysis was performed using two-tailed unpaired *t*-test. Da*t*a were presented as means ± SEM, *n* = 16−18, *P < 0.05, ***P < 0.001, ns not significant.

Next, we sought to isolate different basal cell populations for further functional characterization. We recognized that the surface molecule CD34, a previously identified marker for multipotent hematopoietic stem cells^35^, specifically expressed in the TEB basal cells at both transcription level and protein level (Supplementary Fig. S4a, b). CD34 staining on the FACS plot showed heterogeneous cells in the basal population (Supplementary Fig. S4c). To better sub-fractionate the mammary basal cells, we then introduced another cell surface marker CD200, which was reported to be expressed on the stem cells of various tissues^36–38^. In mammary epithelial cells, we found that CD200 was specifically expressed in ductal/nipple basal cells rather than TEB basal cells (Supplementary Fig. S4d, e). In addition, some of the luminal cells were also CD200^+^ (Supplementary Fig. S4d, e). The mammary stem cells were enriched in CD200^+^ basal population determined by colony-forming assay and transplantation assay (Supplementary Fig. S4f–i). When CD34 staining was combined with CD200, three populations of basal cells can be distinguished on the FACS plot as CD200^−^CD34^+^, CD200^+^CD34^low^, CD200^+^CD34^−^ (Fig. 2a). CD200^−^CD34^+^ cells constituted the majority of TEB basal cells, CD200^+^CD34^−^ cells constituted the majority of nipple basal cells, and duct basal cells were mainly composed of CD200^+^CD34^low^ and CD200^+^CD34^−^ cells (Fig. 2b, c). The three basal populations defined by CD200 and CD34 also express the TEB, duct and nipple signature genes accordingly (Supplementary Fig. 4j, k).

In vitro colony formation assay^16^ was then performed to characterize the proliferation capacity of different basal populations. On day 4, CD200^−^CD34^+^ basal cells quickly established visible colonies on the matrigel, earlier than the other two populations (Fig. 2d, e). However, on day 7, CD200^+^CD34^low^ and CD200^+^CD34^−^ basal cells quickly caught up and formed larger colonies (Fig. 2d, e). This suggests that CD200^−^CD34^+^ basal cells might be fast proliferating cells in the cycle, reminiscent of the TEB basal cell phase shown in Fig. 1f, while CD200^+^CD34^low^ and CD200^+^CD34^−^ basal cells dwelling in duct and nipple basal populations (Fig. 2b, c) and mostly resting in G0 phase (Fig. 1f), were activated later but possessed higher potential in proliferation. This phenotype holds true for the basal cells isolated from nipple, duct and TEB region (Supplementary Fig. S5a, b). In the limiting dilution transplantation assay, the CD200^+^CD34^−^ basal cells exhibited the highest repopulating ability with a stem cell frequency of 1 out of 28.2 (confidence interval 1/11−1/73.6), while CD200^−^CD34^+^ basal cells have the lowest stem cell frequency of 1 out 490 (confidence interval 1/225−1/1071) (Fig. 2f). Secondary transplantation assay confirmed the highest long-term self-renewal ability of the CD200^+^CD34^−^ basal cells (Fig. 2g–j). These data collectively suggests that CD200^+^CD34^−^ basal cells in the nipple region, were enriched for mammary repopulating units. Since ~80% of the basal cells in the nipple region are CD200^+^CD34^−^ as quantified by FACS analysis (Fig. 2b, c), and our transplantation data (Fig. 2f, j) indicate that this population is enriched for mammary stem cells, it suggests that the nipple region has a higher frequency of stem cells than other regions. In contrast, the features of CD200^−^CD34^+^ basal cells enriched in TEB fit better with the concept of progenitor cells with fast proliferation but lower reconstitution capacity.

### *Bcl11b* labels bipotent mammary stem cells in embryonic and postnatal mammary gland

**Fig. 3:**
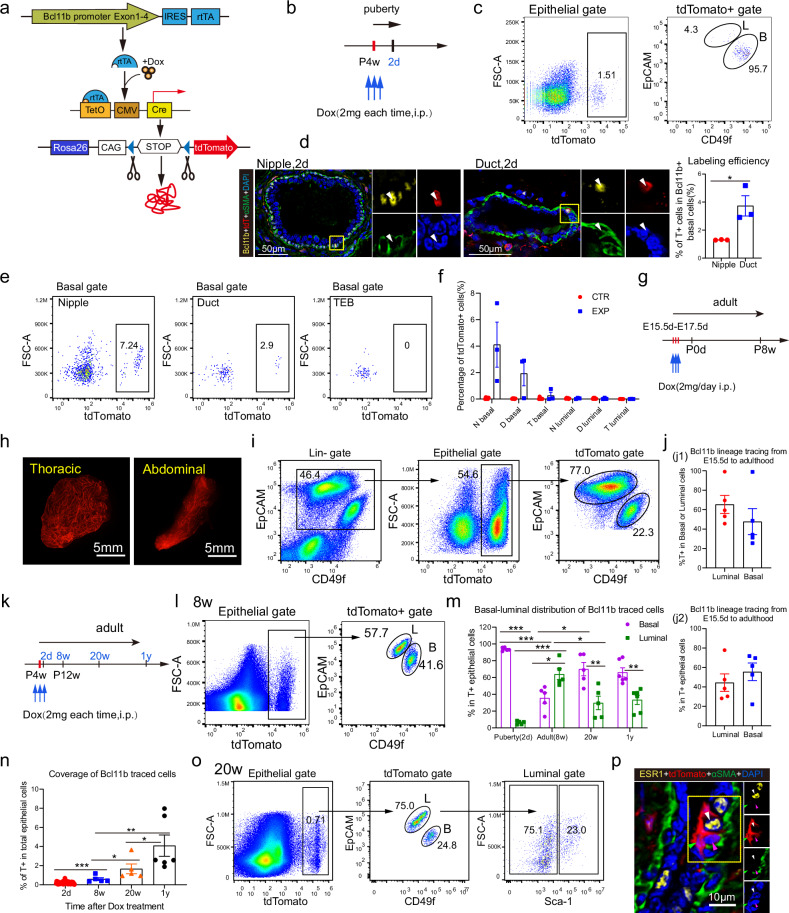
*Bcl11b* labels a group of bipotent mammary stem cells. **a** Schematic diagram of doxycycline-inducible *Bcl11b-rtTA/TetO-Cre/R26-tdTomato* model. *Bcl11b-rtTA* mice were crossed with *TetO-Cre* mice and *R26-tdTomato* reporter mice to generate doxycycline inducible reporter mouse model for Bcl11b^+^ cell lineage tracing. **b** Schematic strategy for lineage tracing Bcl11b^+^ cells from puberty stage. *Bcl11b-rtTA/TetO-Cre/tdTomato* mice were administered with 2 mg/mouse doxycycline (100 µL, 20 mg/mL in PBS) intraperitoneally for three consecutive days at 4 weeks of age. The mammary glands were harvested for tdTomato labeling analysis 2 days after pulsing. **c** Representative FACS plots showing the percentage of tdTomato-positive cells in epithelial population (left panel) and the proportion of basal or luminal subpopulation in tdTomato^+^ epithelial cells (right panel), 2 days after doxycycline induction at puberty. **d** Representative immunofluorescence images showing the co-staining of Bcl11b, tdTomato and basal cell marker αSMA in nipple and duct in mammary glands 2 days after doxycycline induction at puberty (left panel). Yellow, Bcl11b; red, tdTomato; green, αSMA; blue, DAPI. Scale bars, 50 μm. And bar chart showing the labeling efficiency of Bcl11b^+^ cells by tdTomato in nipple and duct of mouse mammary glands (right panel). Statistical analysis was performed using two-tailed unpaired *t* test. Data were presented as means ± SEM, *n* = 3. *P < 0.05. **e** Representative FACS plots showing the tdTomato-labeling percentage in basal cells (Lin^−^Epcam^low^ CD49f^+^) of TEB, duct or nipple 2 days after doxycycline induction from puberty stage. *n* = 3. **f** Bar graph showing the statistical analysis of the percentage of tdTomato^+^ cells in basal or luminal compartments from TEB, duct and nipple 2 days after doxycycline induction from 4-week-old mice by FACS as in **e**. *Bcl11b-rtTA/TetO-Cre/ tdTomato* mice with PBS treatment were control group (CTR), while that with doxycycline treatment were experimented group (EXP). Statistical analysis was performed using two-tailed unpaired *t*-test. Da*t*a were presented as means ± SEM, *n* = 3−5. **g** Schematic diagram showing the lineage tracing strategy of Bcl11b^+^ cells from embryonic day 15.5 in **h**−**j**. **h** Representative whole-mount images of tdTomato-labeled mammary glands in 8-week-old mice traced from embryonic stage. Scale bars, 5 mm. **i** Representative FACS plots display the percentage of tdTomato-positive cells in epithelial population (middle panel), and the proportion of basal or luminal subpopulation in tdToamto^+^ epithelial cells (right panel), from 8-week-old mice traced from embryonic stage. **j** Bcl11b-lineage tracing from the embryonic stage (E15.5 d) to adulthood (P8w). Bcl11b^+^ epithelial cells were traced from embryonic day 15.5 (E15.5 d). When these offspring reached 8 weeks of age (P8w), their mammary glands were harvested for tdTomato-labeling analysis. **j1**, **j2** Bar graphs showing the percentage of tdTomato-labeled cells in luminal or basal population (**j1**) and the percentage of luminal or basal cells among the total tdTomato-labeled epithelial cells (**j2**). Statistical analysis was performed using two-tailed unpaired *t*-test. Da*t*a were presented as means ± SEM, *n* = 5. **k** Schematic diagram showing the lineage-tracing strategy of Bcl11b^+^ cells for induction in pubertal mice at 4 weeks of age, with various chase periods as indicated (2 days, 8 weeks, 20 weeks, 1 year) in **l**−**p**. **l** Representative FACS plots depict the distribution of tdTomato-traced cells in epithelial gate, 8 weeks after induction at puberty. **m,** **n** Bar charts showing the composition of tdTomato-labeled epithelial cells (**m**), the percentage of tdTomato-labeled epithelial cells in total epithelial cells (**n**) in Bcl11b-lineage tracing at designated tracing times after pulsing at puberty. Statistical analysis was performed using two-tailed unpaired *t*-test. Da*t*a were presented as means ± SEM, *n* = 14 (2-day chase), *n* = 5 (8-week chase), *n* = 5 (20-week chase) and *n* = 6 (1-year chase). *P < 0.05, **P < 0.01 and ***P < 0.001. **o** FACS plots showing the contribution of Bcl11b-traced cells to various mammary populations 20 weeks after pulsing at puberty. Sca-1 was used as a marker of the ESR1^+^ luminal cells. **p** Representative immunofluorescence image showing tdTomato-labeled clone containing basal cell (magenta arrowhead), ESR1^+^ luminal cell (white arrowhead) and ESR1^−^ luminal cell (green arrowhead) 20 weeks after pulsing at puberty. Scale bar, 10μm.

To assess the physiological contribution of the various basal cells to mammary homeostasis, we sought to perform the lineage tracing analysis for these cells. We found that Bcl11b expression is enriched in CD200^+^CD34^−^ basal cells at the transcription level (Supplementary Fig. S6a) and in the nipple basal cells at protein level (Supplementary Fig. S3f, g). We previously reported that Bcl11b^high^ basal cells in mouse mammary gland are quiescent mammary stem cells in the basal population, and they have long-term self-renewal ability in the transplantation assay^16^. However, it remains unclear how Bcl11b^high^ basal cells behave under the physiological condition. We therefore generated the *Bcl11b* tracing mouse by employing genetic engineering techniques to insert an ‘IRES-rtTA’ cassette at the end of *Bcl11b* coding region (Fig. 3a) without disturbing the expression level of Bcl11b and relative mammary epithelial signature genes (Supplementary Fig. S6b–d). After crossing with *TetO-Cre* mouse and tdTomato reporter mouse, we were able to label and trace Bcl11b-positive cells by doxycycline induction (Fig. 3; Supplementary Fig. S9a–d). After a brief pulse labeling in the pubertal mammary gland, Bcl11b-positive mammary cells can be clearly identified in the basal population (an average of 93.7% ± 2.0% labeled epithelia were basal, *n* = 5) in the FACS analysis (Fig. 3b, c, m) and in the immunofluorescence staining analysis (Fig. 3d). The pulse labeled cells were confirmed to highly express Bcl11b at the transcription level (Supplementary Fig. S6e, f). Moreover, the Bcl11b-traced cells were preferentially accumulated in nipple-derived basal cells, with gradually decreasing percentages in ducts and TEBs (Fig. 3e–f). However, we noticed that these Bcl11b^+^ quiescent cells were difficult to be efficiently labeled, because the labeled cell frequency was significantly lower than expected from immunofluorescence staining in nipple structure even at very high doxycycline dose with various pulsing strategies (Fig. 3d; Supplementary Fig. S6g, h). This is possibly due to the quiescence nature of Bcl11b^+^ cells (Supplementary Fig. S6i).

To ask what progenies Bcl11b-positive cells give rise to during mammary development and homeostasis, we employed various tracing strategies. When Bcl11b-positive cells were labeled in embryonic mammary gland at day E15.5–E17.5 and were analyzed at adult stage (Fig. 3g, h; Supplementary Fig. S6k), a significant amount of mammary cells including basal and luminal cells were traced (Fig. 3i, j), suggesting that embryonic Bcl11b-positive cells are bipotent mammary stem cells. When we pulse labeled Bcl11b^+^ mammary cells during puberty at week 4 and analyzed after 8 weeks at adult stage (Fig. 3k), we found that the Bcl11b-positive basal cells also gave rise to both luminal and basal cells (Fig. 3l), suggesting that the pubertal Bcl11b-positive basal cells are also bipotent. The progeny of Bcl11b-positive cells formed discrete small clones in the mammary gland, which were composed of basal cells, ER^+^ luminal cells and ER^−^ luminal cells (Fig. 3p). In addition, extended tracing period up to one year showed that the bipotency of Bcl11b^+^ cells was well maintained with the ability to contribute to basal cells, Sca1^+^ (ER^+^) and Sca1^−^ (ER^−^) luminal cells^39,40^ in the FACS analysis (Fig. 3m–o). The percentage of Bcl11b progeny cells in the mammary epithelia kept increasing during the extended tracing period (Fig. 3m, n). These Bcl11b bipotent mammary stem cells persisted in the adult stage, as the pulse labeling at 6 months with one year tracing resulted in prominent bi-lineage contribution to the progeny (Supplementary Fig. S6l, m), which was not due to the leakiness of the mouse model (Supplementary Fig. S6n–s). These data collectively demonstrate that Bcl11b-positive cells are rare mammary stem cells that are difficult to label but have rigorous multipotency even in the postnatal mammary gland.

### *Sema3a* labels a group of unipotent basal progenitor cells

**Fig. 4:**
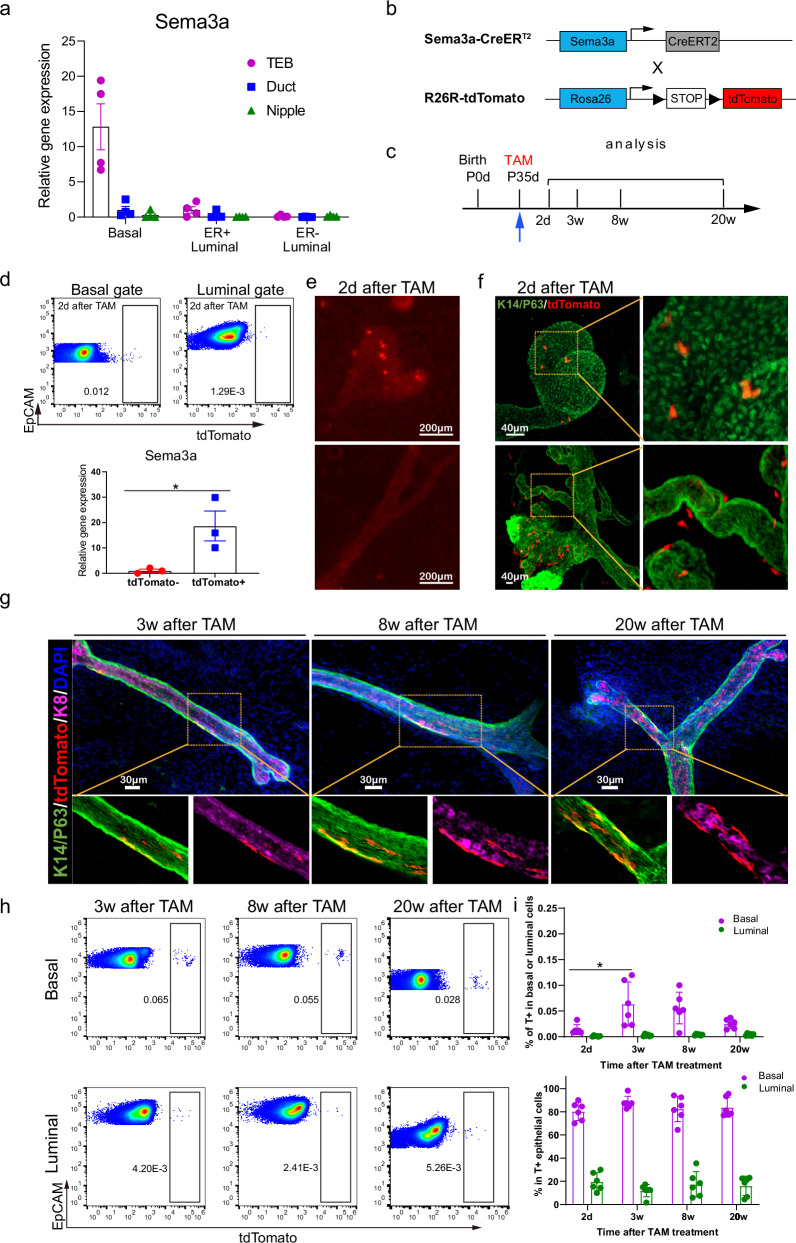
*Sema3a* labels a group of unipotent mammary basal progenitor cells. **a** Real-time PCR analysis showing mRNA level of *Sema3a* in basal, ESR1^+^ luminal, or ESR1^−^ luminal cells from TEB, duct or nipple of puberty mice. Statistical analysis was performed using two-tailed unpaired *t*-test. Data were presented as means ± SEM, *n* = 4. **b** Schematic diagram of tamoxifen-inducible *Sema3a-CreERT*2/*R26R-tdTomato* mouse model. *Sema3a-CreERT2* mice were crossed to *Rosa26-tdTomato* mice to generate tamoxifen inducible reporter mice for Sema3a^+^ cell lineage-tracing. **c** Schematic diagram illustrating the lineage-tracing strategy for Sema3a^+^ cells from puberty for experiments in **d**−**i**. *Sema3a-CreERT2*/*R26-tdTomato* mice were treated with 5 mg tamoxifen (TAM) intraperitoneally for only once at 5-week-old. Mammary glands of various chase periods (2 days, 3 weeks, 8 weeks, 20 weeks) were harvested for tdTomato labeling analysis. **d** Representative FACS plots showing percentage of tdTomato-labeled basal or luminal cells 2 days after pulsing on postnatal day 35 (upper panel). Real-time PCR analysis showing mRNA level of *Sema3a* in tdTomato-labeled and unlabeled basal cells (lower panel). Statistical analysis was performed using two-tailed unpaired *t*-test. Data were presented as means ± SEM, *n* = 3. *P < 0.05. **e** Representative images taken by fluorescence stereomicroscope display tdTomato expression in TEB (upper panel) and duct (lower panel) 2 days after tamoxifen induction at puberty. Scale bars, 200 μm. **f** Representative tissue clearing images showing immunofluorescence staining of tdTomato^+^ cells with basal markers Krt14 and Trp63 in TEB (upper panel) or duct/nipple (lower panel) 2 days after tamoxifen induction. The selected regions by the yellow boxes were magnified in the right panel. Green, Krt14/Trp63; red, tdTomato; blue, DAPI. Scale bars, 40 μm. **g** Representative images showing 3D immunofluorescence staining of tdTomato, basal markers Krt14 and Trp63, and luminal marker Krt8 in tissue cleared mammary gland at different time points after tamoxifen induction at puberty. Green, Krt14/Trp63; red, tdTomato; magenta, Krt18; blue, DAPI. Scale bars, 30 μm. **h** Representative FACS plots showing percentage of tdTomato-labeled basal or luminal cells at the indicated time points after pulsing at puberty. **i** The upper bar graphs showing the percentage of tdTomato-positive cells in basal or luminal compartment at different time points after pulsing at puberty, meanwhile the lower panel showing the relative basal and luminal percentage in tdTomato-positive mammary epithelial cells. Statistical analysis was performed using two-tailed unpaired *t*-test. Da*t*a were presented as means ± SEM, *n* = 6. *P < 0.05.

Previous studies reported that Krt14^+^ cells in the postnatal mammary gland are predominantly unipotent in the lineage tracing assay^8,10,14,18,28^. However, the molecular identity of the unipotent stem cells is still not clear. We therefore tried to lineage trace the CD200^−^CD34^+^ basal cells, a potential mammary progenitor population enriched in the terminal end buds and characterized their physiological behavior in mammary homeostasis. We noticed that *Sema3a*, a cell marker that was used to trace Purkinje fibers in heart^41^, was specifically highly expressed in CD200^−^ basal cells in our initial screening of the gene targets (Supplementary Fig. S5c). Further validation indicated that Sema3a was also highly expressed in TEBs (Fig. 4a; Supplementary Fig. S5d) and part of CD200^−^CD34^+^ basal cells (Supplementary Fig. S4j). Thus, we used the *Sema3a-CreERT2/R26R-tdTomato* mice and treated them with tamoxifen at postnatal day 35 and then followed up the fate of the tdTomato-labeled cells in mammary glands (Fig. 4b, c; Supplementary Fig. S7a–c). Pulse labeling of Sema3a-positive cells confirmed their RNA expression in tdTomato-positive cells (Fig. 4d) and showed clear basal cell identity (Krt14^+^Trp63^+^Krt8^−^) and TEB specificity in the mammary epithelia (Fig. 4e, f). We also identified tdTomato^+^ cells in the duct and nipple region, but these cells were mainly stromal rather than the epithelial cells (Fig. 4f). After chasing for 3 weeks, 8 weeks and 20 weeks, the progenies of Sema3a-positive cells formed clones in the mammary tree, which were exclusively restricted in the basal layer (Fig. 4g). FACS analysis of the Sema3a-traced cells confirmed this basal restricted distribution (Fig. 4h, i). To further confirm the long-term contribution of Sema3a^+^ cells to mammary gland, we performed serial transplantation of tdTomato^+^ clones and observed long lasting contribution of Sema3a^+^ cells to basal population (Supplementary Fig. S7d–g). These data suggest that Sema3a^+^ basal cells in the pubertal mammary gland are a specific basal population distinct from bipotent mammary stem cells and are predominantly unipotent which contribute to the basal lineage for long term.

### Bcl11b^+^ bipotent and Sema3a^+^ unipotent mammary stem cells contribute to mammary reproductive cycles with different kinetics

**Fig. 5:**
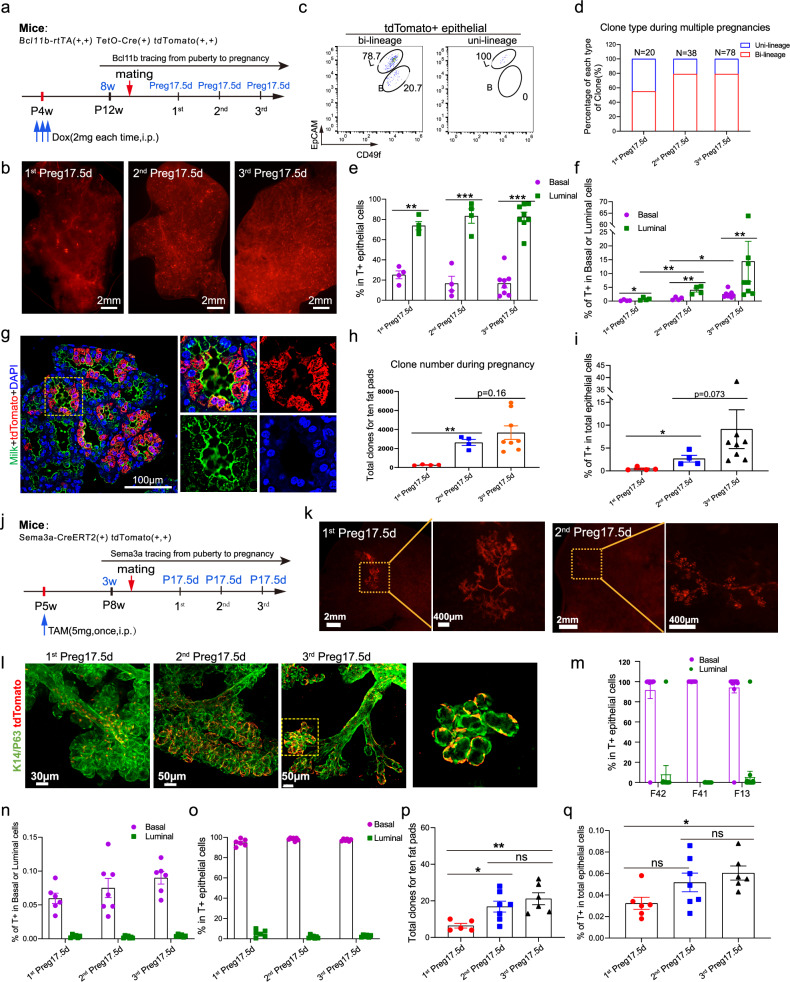
Bcl11b^+^ bipotent and Sema3a^+^ unipotent mammary stem cells contribute to mammary reproductive cycles with different kinetics. **a** Schematic diagram showing the lineage-tracing strategy of Bcl11b^+^ cells from puberty for experiments in **b**−**i**. *Bcl11b-rtTA (+, +) TetO-Cre (+) tdTomato (+, +)* female mice were administered with 2 mg doxycycline intraperitoneally for three consecutive days at 4-week-old, and then mated during adult stage for multi-pregnancy. Mammary glands at various time points as indicated during multiple pregnancies (1^st^ Preg17.5 d, 2^nd^ Preg17.5 d, 3^rd^ Preg17.5 d) were harvested for tdTomato labeling analysis. **b** Representative fluorescence images showing tdTomato-positive clones in mammary glands of *Bcl11b-rtTA/TetO-Cre/tdTomato* mice at the indicated time points during multiple pregnancies after pulsing at puberty. **c** Representative FACS plot showing the composition of tdTomato-positive cells in two types of individual clones (bi-lineage in the left panel and uni-lineage in the right panel) in Bcl11b-lineage tracing during multiple pregnancies (1^st^ to 3^rd^ pregnancy) after pulsing at puberty. The individual bright tdTomato^+^ Bcl11b clones in the mammary fat pads during multiple pregnancies were dissected under the fluorescence stereomicroscope, and then were digested into single cells and performed FACS analysis of the composition of basal and luminal lineages. **d** Bar chart showing percentage of bi-lineage and uni-lineage clones at day 17.5 of multiple pregnancies (1^st^ to 3^rd^ Preg17.5 d) in *Bcl11b-rtTA/TetO-Cre/tdTomato* mice after pulsing at puberty. As for counted clones, *n* = 20 for 1^st^ Preg17.5 d, *n* = 38 for 2^nd^ Preg17.5 d, *n* = 78 for 3^rd^ Preg17.5 d, and the clones of each group were collected from at least 3 mice. **e**, **f** Bar charts showing the composition of tdTomato-labeled epithelial cells (**e**), and percentage of tdTomato-labeled cells in basal or luminal populations (**f**) at the indicated time points during multiple pregnancies in Bcl11b-lineage tracing after pulsing at puberty. Data were presented as means ± SEM, *n* = 4−8. **P < 0.01, ***P < 0.001. **g** Representative images showing milk production by Bcl11b^+^ cells derived epithelial cells at lactation day 7 in Bcl11b-lineage tracing after pulsing in puberty. The selected region by the yellow frame was enlarged in the right panel. Green, milk; red, tdTomato; blue, DAPI. Scale bars, 100 μm. **h** Bar chart showing the number of tdTomato-labeled clones as shown in **b** in all ten fat pads of each mouse at the indicated time points during multiple pregnancies in Bcl11b-lineage tracing after pulsing at puberty. Data were presented as means ± SEM, *n* = 4 − 8. **P < 0.01. **i** Bar chart showing percentage of tdTomato-labeled cells in all epithelial cells at the indicated times during multiple pregnancies in Bcl11b-lineage tracing after pulsing at puberty. Data were presented as means ± SEM, *n* = 4−8. *P < 0.05. **j** Schematic diagram showing the lineage-tracing strategy of Sema3a^+^ cells from puberty for experiments in **k**−**q**. *Sema3a-CreERT2 (+) tdTomato (+, +)* female mice were treated with single time 5 mg tamoxifen intraperitoneally at 4-week-old, and then mated during adult stage for multi-pregnancy. Mammary glands at various time points as indicated during multiple pregnancies (1^st^ Preg17.5 d, 2^nd^ Preg17.5 d, 3^rd^ Preg17.5 d) were harvested for tdTomatoto labeling analysis. **k** Representative fluorescence images of tdTomato-positive clones in mammary gland at pregnancy day 17.5 during the 1^st^ or 2^nd^ pregnancies in Sema3a-lineage tracing after pulsing at puberty. The selected regions by the yellow frames were enlarged in the right panel. Scale bars, 400 μm or 2 mm. **l** Representative 3D immunofluorescence image of tissue cleared mammary gland showing the co-staining of tdTomato with basal cell markers Krt14 and Trp63 in mammary glands of *Sema3a-CreERT2*/*tdTomato* mice during multiple pregnancies after pulsing at puberty. The selected region by the yellow box was magnified in the right panel. Green, Krt14/Trp63; red, tdTomato. Scale bars, 30 μm or 50 μm. **m** Bar graph showing the composition of tdTomato-labeled epithelial cells of individual clones at day 17.5 of the 1^st^ pregnancy from each mouse with Sema3a-lineage tracing at puberty. Each dot represents one clone from a mouse whose ear tag number was labeled below the corresponding bars. Data were presented as means ± SEM, *n* = 6 − 7. **n**, **o** Bar charts showing percentage of tdTomato-labeled cells in basal or luminal populations (**n**), and basal or luminal proportion in all tdTomato-labeled epithelial cells (**o**) at the indicated time points during multiple pregnancies in Sema3a-lineage tracing from puberty. Data were presented as means ± SEM, *n* = 6–7. **p** Bar chart showing the number of tdTomato-labeled clones in all ten fat pads of each mouse at the indicated time points during multiple pregnancies in Sema3a-lineage tracing after pulsing at puberty. Data were presented as means ± SEM, *n* = 5−7. *P < 0.05, **P < 0.01, ns, not sig*n*ificant. **q** Bar chart showing the percentage of tdTomato-labeled cells in all epithelial cells at the indicated time points during multiple pregnancies in Sema3a-lineage tracing after pulsing at puberty. Data were presented as means ± SEM, *n* = 6−7. *P < 0.05, ns, not significant. All above statistical analysis was performed using two-tailed unpaired *t*-test.

Our data have shown that the bipotent and unipotent mammary stem cells are distinct populations that co-exist in the postnatal mammary gland. Next, we asked why mammary gland needs various types of stem cells to maintain the mammary structure and function. One possibility is that during reproduction cycles when massive mammary cell expansion is required, diverse mammary stem cells might be imperative to assure not only the efficient mammary reorganization, but also a sustainable stem cell/progenitor pool. We therefore pulse labeled Bcl11b-positive cells during puberty and analyzed the progenies at pregnancy day 17.5 (Preg 17.5 d) (Fig. 5a). Small clones at limited foci were identified at the first pregnancy (Fig. 5b), and these clones were spatially accumulated in the middle region of the gland (Supplementary Fig. S8a, b). This might result from the fact that Bcl11b-positive cells were predominantly quiescent in the nipple region while the Bcl11b-positive cells in the middle region were relatively active and able to expand into discrete clones. Further analysis showed that 55% of the individual clones (1^st^ pregnancy,11 of 20) were bi-lineage (Fig. 5c, d) and Bcl11b-positive cells preferentially gave rise to luminal cells during pregnancy (Fig. 5e, f; Supplementary Fig. S9a, c, d), possibly due to the faster proliferation rate for luminal cells. In addition, the Bcl11b^+^ cell-derived alveolar structures were able to lactate and functionally contribute to the mammary gland (Fig. 5g). Most intriguingly, when we performed multiple rounds of pregnancy, tdTomato-positive cells exhibited significant increase of the clone number, the percentage of bi-lineage clones (79% (30 of 38) for the 2^nd^ pregnancy and 80.1% (63 of 78) for the 3^rd^ pregnancy), as well as the mammary epithelial coverage (Fig. 5b–i). The clonal cell composition analysis by immunofluorescence staining in various tracing schemes reinforces the conclusion of the bipotency and clonal expansion of Bcl11b^+^ cells (Supplementary Figs. S8d-j, 9d). These data suggest that Bcl11b-positive stem cells are long-term survivors with accumulating contributions to mammary maintenance.

In contrast, Sema3a-labeled unipotent stem cells exhibit different kinetics in lineage contribution during pregnancy. When we pulse labeled Sema3a-positive cells during puberty and analyzed at Preg17.5 d (Fig. 5j), large clones were identified with preferential distribution at the distal part of the mammary fat pad (Fig. 5k; Supplementary Fig. S8a, c). The traced clones were predominantly basal cells as revealed by 3D cleared tissue imaging analysis (Fig. 5l; Supplementary Fig. S8k–m) and by the FACS analysis for individual clones (Fig. 5m) and for the whole mammary gland (Fig. 5n, o). However, different from bipotent mammary stem cells, the unipotent mammary stem cell clone number and epithelial coverage remained relatively stable after multiple rounds of pregnancy (Fig. 5p, q). These data suggest that there are different mammary stem cell populations involved in the development of pregnant mammary gland. And the relative contributions of various mammary stem cells to the gland are dynamically changing during multiple pregnancies.

**Fig. 6:**
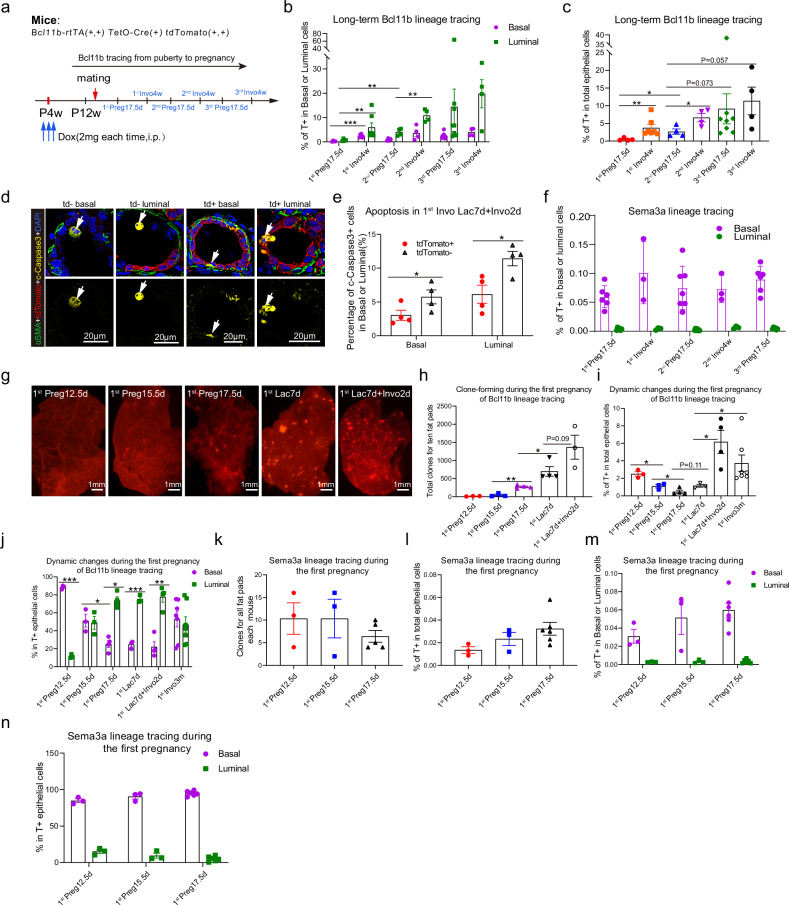
Distinct contributions of Bcl11b^+^ bipotent and Sem3a^+^ unipotent stem cells to the mammary reorganization during pregnancy. **a** Schematic diagram showing the tracing strategy of Bcl11b^+^ cells during multiple pregnancies and involutions. **b**, **c** Bar chart showing the percentage of tdTomato-labeled cells in basal or luminal populations (**b**), or percentage of tdTomato-labeled cells in the whole mammary epithelial population (**c**) at the indicated time points during multiple pregnancies and involutions in Bcl11b-lineage tracing after pulsing at puberty. Data were presented as means ± SEM, *n* = 4−8. **P < 0.01, ***P < 0.001. **d** Representative immunofluorescence images showing the apoptotic cells indicated by cleaved caspase3 staining during involutions for Bcl11b-traced (tdTomato^+^) and -untraced (tdTomato-) cells during mammary involution. Scale bars, 20 μm. **e** Bar chart showing the quantification of apoptotic cells in Bcl11b-traced (tdTomato^+^) and -untraced (tdTomato-) cells during mammary involution. Data were presented as means ± SEM, *n* = 4. *P < 0.05. **f** Bar chart showing percentage of tdTomato-labeled cells in basal or luminal populations at the indicated time points during multiple pregnancies and involutions in Sema3a-lineage tracing after pulsing at puberty. Data were presented as means ± SEM, *n* = 3–7. **g** Representative images of tdTomato-positive clones in mammary glands at day 12.5, 15.5 or 17.5 of the 1^st^ pregnancy (1^st^ Preg12.5 d, 15.5 d, 17.5 d), day 7 of the 1^st^ lactation (Lac7d) and involution day 2 (Invo2d) in Bcl11b-lineage tracing after pulsing at puberty. Scale bars, 1 mm. **h** Bar chart showing the number of tdTomato-positive clones as shown in **g** in all ten fat pads of each mouse at the indicated time points of the 1^st^ reproductive cycle in Bcl11b-lineage tracing after pulsing at puberty. Data were presented as means ± SEM, *n* = 3−4. *P < 0.05, **P < 0.01. **i**, **j** Bar charts showing percentage of tdTomato-labeled cells in total epithelial population (**i**) and basal and luminal proportion in all tdTomato-labeled epithelial cells (**j**) at the indicated time points during the 1^st^ reproductive cycle in Bcl11b-lineage tracing after pulsing at puberty. ‘Invo 3 m’ is designated as 90 days after involution. Data were presented as means ± SEM, *n* = 3−7. *P < 0.05, **P < 0.01, ***P < 0.001. **k** Bar chart showing the tdTomato-labeled clone number in all ten fat pads of each mouse at the indicated time points during the 1^st^ pregnancy in Sema3a-lineage tracing after pulsing at puberty. Data were presented as means ± SEM, *n* = 3−5. **l**–**n** Bar charts showing percentage of tdTomato-labeled cells in the whole mammary epithelial population (**l**), percentage of tdTomato-labeled cells in basal or luminal subpopulations (**m**), and basal or luminal proportion in all tdTomato-labeled epithelial cells (**n**) at the indicated time points during the 1^st^ pregnancy in Sema3a-lineage tracing after pulsing at puberty. Data were presented as means ± SEM, *n* = 3−6. All above statistical analysis was performed using two-tailed unpaired *t* test.

Since we have observed a clear increase of the clone number (Fig. 5b, h) and the coverage of Bcl11b progenies in multiple rounds of pregnancies (Fig. 5f, i), in contrast to other lineage tracing report^18^, we next asked why clone number expanded significantly for bipotent mammary stem cells between pregnancies. We also wanted to clarify when the increase of the coverage of Bcl11b progeny occurs and what is the trigger. Then we analyzed the involuted mammary gland in the lineage tracing experiment. We found that the increase of the epithelial coverage mainly occurred specifically after each round of involution for Bcl11b^+^ bipotent mammary stem cells in Bcl11b tracing (Fig. 6a–c), suggesting that the progeny of bipotent stem cells persisted better than the rest mammary cells during mammary regression. Moreover, apoptosis analysis of involuted mammary gland showed that the Bcl11b-traced tdTomato^+^ cells were less apoptotic compared with tdTomato^−^ cells (Fig. 6d, e). In contrast, Sema3a-traced progeny of unipotent mammary stem cells did not show this trend (Fig. 6f). These data suggest that the increase of the coverage is due to the higher survival rate of Bcl11b progenies against apoptosis during involution.

To ask how Bcl11b^+^ and Sema3a^+^ cells behave differently during pregnancy at an improved time resolution, we analyzed tdTomato-labeled cells at pregnancy days 12.5, 15.5 and 17.5 (Supplementary Fig. S9a). We found that the clone number derived from Bcl11b^+^ bipotent mammary stem cells increased from pregnancy day 12.5 to day 17.5 (Fig. 6g, h); however, the coverage of Bcl11b^+^ bipotent mammary stem cell progeny showed a decrease during pregnancy day 12.5 to day 17.5, followed by an increase during early lactation (Fig. 6g–j). This suggests that although Bcl11b-traced cells are expanding their population, they were not as proliferative and competitive as the rest of mammary cells. Indeed, for Sema3a unipotent mammary stem cell traced cells, even though the clone number kept stable, the coverage in epithelial cells showed an increasing trend during the same period (Fig. 6k–n). These data collectively indicate that different mammary stem cells respond to the pregnancy cues in different modes.

We showed that Bcl11b^+^ cells were enriched in the nipple region with lower proliferation index, and Sema3a^+^ cells were enriched in TEBs with higher proliferation index (Fig. 6i, l; Supplementary Figs. S1d, 2c, 3f, 3i, 4k, 5c, 6i). These data suggest that Bcl11b^+^ cells normally stay in less active state and need signaling molecules to activate their proliferation. Therefore, in the lineage tracing assay during pregnancy, it takes longer time for Bcl11b^+^ cells to exit from its quiescent state and enter an active state, compared with Sema3a^+^ cells. From this perspective, Bcl11b^+^ cells can be regarded as slow responders. However, after Bcl11b^+^ cells activation, they start to proliferate and differentiate into luminal cells. Within Bcl11b progenies, as luminal cells proliferate faster than basal cells, the luminal basal ratio cannot be maintained as 1:1, luminal cells outcompete basal cells and gradually form a predominant population (Fig. 6b, f, j, n). Thus, Bcl11b^+^ mammary stem cells are more like a slow responder but persist for longer time and gradually replenish the mammary gland, while the Sema3a^+^ mammary stem cells respond more rapidly to pregnancy but have restricted contributions to the gland in long term.

### Bcl11b is indispensable for long term maintenance of bipotent mammary stem cells

**Fig. 7:**
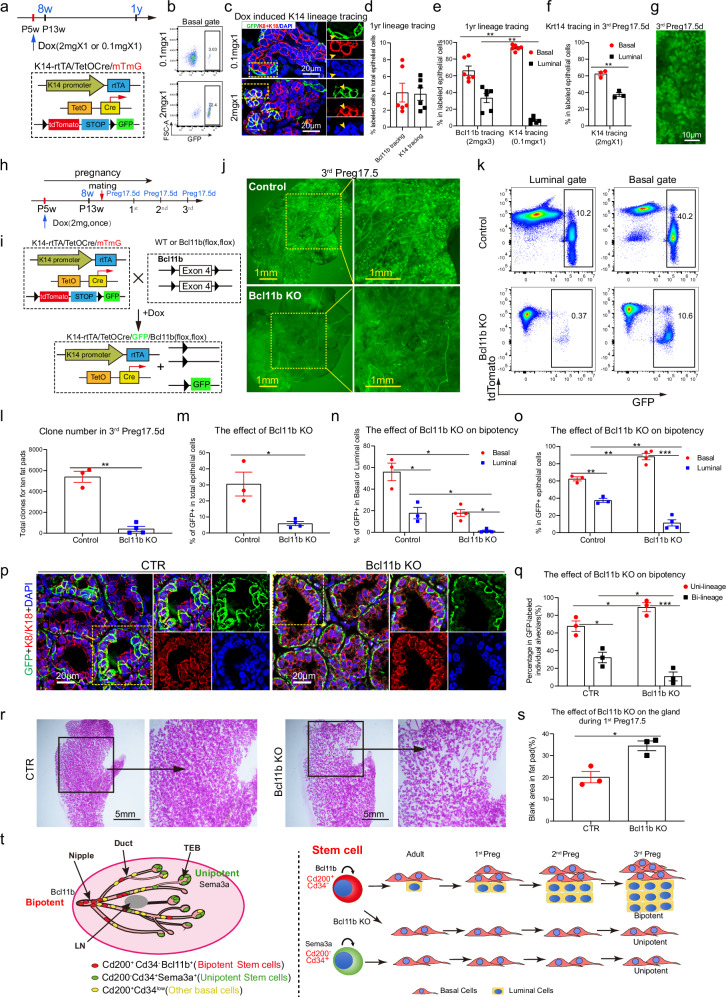
Bcl11b is indispensable for mammary stem cell bipotency. **a** The upper panel shows schematic diagram of doxycycline-inducible *Krt14-rtTA/TetO-Cre/R26-mTmG* model. *Krt14-rtTA* mice were crossed with *TetO-Cre* mice and *R26R-tdTomato* reporter mice to generate doxycycline inducible reporter mouse model for lineage tracing Krt14^+^ cell. The lower panel shows schematic strategy for lineage tracing studies of Krt14^+^ cells from puberty stage for experiments in **b**−**d**. *Krt14-rtTA/TetO-Cre/R26-mTmG* mice were administered with 0.1 mg or 2 mg doxycycline intraperitoneally only once at postnatal 5-week-old (P5w). The mammary glands were harvested for GFP labeling analysis 1 year after pulsing. **b** Representative FACS plots showing the labeling efficiency of mammary basal cells after doxycycline treatment at a low (0.1 mg) or high (2 mg) dose for 2 days. **c** Representative images showing immunofluorescence co-staining of GFP and luminal marker Krt8/18 in mammary glands of Krt14 tracing 1 year after pubertal pulsing with a single low dose of 0.1 mg (upper panel) or a high dose of 2 mg (lower panel). The selected regions by the yellow boxes were magnified in the right panel. Yellow arrowheads indicate the GFP-traced cells not co-localized or co-localized with Krt8/18 labeling respectively with low or high doxycycline induction. Green, GFP; red, K8/K18; blue, DAPI. Scale bars, 20 μm. **d** Bar chart showing the similar percentage of tracing-reporter labeled cells in the whole mammary epithelial population 1 year after doxycycline pulsing at puberty, tdTomato reporter for Bcl11b-lineage tracing (2 mg doxycycline) and GFP reporter for Krt14-lineage tracing (0.1 mg doxycycline). Pulsing methods are annotated below, and data were presented as means ± SEM, *n* = 6, ns, not significant. **e** Bar chart showing the composition of the Bcl11b-traced and Krt14-traced epithelia 1 year after pubertal pulsing in Bcl11b- and Krt14-lineage tracing. Pulsing methods are annotated below, and data were presented as means ± SEM, *n* = 6. **P < 0.01. **f** Bar chart showing the composition of Krt14 traced epithelial cells at day 17.5 of the 3^rd^ pregnancy after pubertal pulsing at a high dose of doxycycline 2 mg per mouse. Data were presented as means ± SEM, *n* = 3. **P < 0.01. **g** Fluorescence image showing the Krt14-traced positive clones in the mammary gland at day 17.5 of the 3^rd^ pregnancy after pubertal pulsing at a high dose of doxycycline 2 mg per mouse. Scale bar, 10 μm. **h**, **i** Schematic diagram showing the lineage tracing strategy for Krt14-positive cells from puberty in the presence of WT or KO of *Bcl11b* for experiments in **j**−**s**. Specifically, *Krt14-rtTA/TetO-Cre/mTmG*/*Bcl11b*^*wt/wt*^ or *Krt14-rtTA/TetO-Cre/mTmG*/*Bcl11b*^*flox/flox*^ mice at 5 weeks of age were pulsed by a high dose of doxycycline (2 mg) and then mated during adult stage for multi-pregnancy. Mammary glands chased for various periods of time indicated during multiple pregnancies (1^st^ Preg17.5 d, 2^nd^ Preg17.5 d, 3^rd^ Preg17.5 d) were harvested for analysis of GFP-labeled cells. **j** Representative fluorescence images of GFP-positive clones in mammary glands at day 17.5 of the 3^rd^ pregnancy in control or *Bcl11b* KO group after pulsing at puberty. The selected regions by the yellow boxes were magnified in the right panel. Scale bars, 1 mm. **k** Representative FACS plots showing percentage of GFP-labeled cells in basal or luminal compartments in control or *Bcl11b* KO group at day 17.5 of the 3^rd^ pregnancy after pulsing at puberty. **l** Bar chart showing the number of GFP-positive clones in all ten fat pads of each mouse in control or *Bcl11b* KO group at day 17.5 of the 3^rd^ pregnancy after pulsing at puberty. Data were presented as means ± SEM, *n* = 3−4. **P < 0.01. **m**–**o** Bar charts showing percentage of GFP-labeled cells in the whole epithelial population (**m**), percentage of GFP-labeled cells in basal or luminal subpopulations (**n**), and basal or luminal proportion in all GFP-labeled epithelial cells (**o**) in control or *Bcl11b*-KO group at day 17.5 of the 3^rd^ pregnancy after pulsing at puberty. Data are presented as means ± SEM, *n* = 3−4. *P < 0.05, **P < 0.01, ***P < 0.001. **p** Representative images of GFP-positive clones with immunofluorescence staining of GFP and luminal marker Krt8/18 in mammary glands of control or *Bcl11b*-KO group based on Krt14-lineage tracing at day 17.5 of the 3^rd^ pregnancy after pulsing at puberty. The selected regions by the yellow boxes were split for different channels. Green, GFP; red, Krt8/Krt18; blue, DAPI. Scale bars, 20 μm. **q** Quantification of the uni-lineage or bi-lineage GFP clones judged by immunostaining as shown in **p** from control or *Bcl11b*-KO group at day 17.5 of the 3^rd^ pregnancy after pulsing at puberty. Data were presented as means ± SEM, for mice in each group, *n* = 3; for the counted clones of each mouse, *n* = 600. *P < 0.05, ***P < 0.001. **r** Representative whole-mount carmine staining images of the thoracic mammary glands from control *(Krt14-rtTA/TetO-Cre/mTmG*/*Bcl11b*^*wt/wt*^*)* and *Bcl11b*-KO *(Krt14-rtTA/TetO-Cre/mTmG*/*Bcl11b*^*flox/flox*^*)* group at day 17.5 of the 1^st^ pregnancy after a high dose of doxycycline (2 mg) induction at puberty. The selected regions by the black frames were enlarged in the right panel. Scale bars, 5 mm. **s** Quantification of the blank area without epithelial cells stained by carmine in the thoracic fat pads of control and *Bcl11b*-KO group displayed in **r**. Data were presented as means ± SEM, *n* = 3. *P < 0.05. **t** Schematic diagram showing the existence of both bipotent and unipotent basal stem cells in pubertal mammary glands. The Bcl11b^+^ bipotent stem cells are enriched in a CD200^+^CD34^−^ basal subpopulation and locate densely in nipple while sparsely in duct and TEB. And the Sema3a^+^ unipotent basal stem cells are enriched in a CD200^−^CD34^+^ basal subpopulation and locate specifically in TEB. All above statistical analysis was performed using two-tailed unpaired *t* test.

Our data clearly showed different types of clones formed by bipotent and unipotent mammary stem cells, and we therefore were curious about the biological significance of these two types of morphogenesis. Previous studies by Krt14 tracing revealed variable lineage contributions with controversial multipotency in the postnatal mammary development^18,28^. Here we found that the contribution of the rare Bcl11b^+^ cells to both basal and luminal lineages became more obvious after 1 year tracing or multi-reproductive cycles (Fig. 7a–f). We speculate that Krt14^+^ cells should also contain bipotent mammary stem cells^28^ with similar behavior of Bcl11b^+^ cells. The dosage of the inducer, as proposed previously^5^, could make a difference in tracing experiment. We therefore started to reevaluate the lineage tracing of Krt14 cells for long term (Fig. 7a; Supplementary Fig. S10a) with high (2 mg) and low (0.1 mg) dose doxycycline treatment. A single high dose of doxycycline resulted in more than 70% of basal cells labeled in contrast to 3% for low dose induction group (Fig. 7b). After one year’s tracing, the bi-lineage clones can be clearly identified in high dose group but rarely in low dose group (Fig. 7c; Supplementary Fig. S10l). At similar labeling rate in the one-year tracing assay, Bcl11b^+^ cells showed clear bipotency while Krt14^+^ cells (low dose induction) were mainly unipotent (Fig. 7d, e). It suggests that it is the labeled cell type rather than the labeling rate that determines the tracing outcome. The bipotency of Krt14 (high dose) labeled cells were more prominent in multi-pregnancy tracing (Fig. 7f) and formed discrete small clone foci similar to Bcl11b tracing (Fig. 7g). These small clones were not well characterized in previous studies possibly due to their low labeling efficiency and slow response rate. We speculate that these bipotent small clones in Krt14 tracing might originate from the Bcl11b^+^ bipotent stem cells.

To ask whether Bcl11b is functionally significant for the production of the bipotent mammary clones, we induced the depletion of Bcl11b in Krt14-positive basal cells (Supplementary Fig. S10e) by doxycycline and traced the cells for one year or multiple rounds of pregnancy (Fig. 7h, i). The basal cells were initially labeled by ~70% after initial pulse labeling (Supplementary Fig. S10b–d). After one year tracing, Krt14^+^ control cells gave rise to more luminal cells than *Bcl11b*-knockout (KO) cells (Supplementary Fig. S10f–k). This phenomenon was more prominent in multi-pregnancy tracing assay. After three rounds of pregnancy, GFP^+^ foci were observed in control mammary glands of Krt14-lineage tracing, while a striking clone reduction (12.9-fold) in *Bcl11b*-KO mammary glands (Fig. 7j, l). The mammary epithelial coverage of *Bcl11b*-KO cells was also significantly reduced accordingly (Fig. 7k–m). Most intriguingly, the basal vs luminal ratio of the traced clones shifted from 1.5:1 in control mice to 9:1 in *Bcl11b*-KO mice (Fig. 7n, o; Supplementary Fig. S10m–o). This suggests that loss of Bcl11b in basal cells impairs the bipotency of mammary stem cells and the mutant mammary stem cells tend to be more unipotent. Indeed, when we immune-stained the control and *Bcl11b*-KO mammary gland, bi-lineage clones were identified in control samples but rarely in *Bcl11b*-KO samples (Fig. 7p, q).

To assess the physiological significance of Bcl11b^+^ cells in mammary homeostasis, we analyzed the morphology of mammary gland during pregnancy after *Bcl11b*-KO. Loss of Bcl11b resulted in a sparser mammary tree with increased blank area on the fat pad compared with the control (Fig. 7r, s). Overall, these data demonstrate that mammary glands in adult and reproduction cycles are maintained by both bipotent and unipotent mammary stem cells, which contribute to the integrity of mammary tissue in distinct modes.

## Discussion

Mammary gland lineage hierarchy has been extensively studied in recent years. While stem cells have been widely accepted as a long-lived cell population that sustain mammary architecture and function, their molecular identity, cellular heterogeneity and differentiation trajectory remain obscure. Depending on the experimental strategies, various stem cell^8–11,14,17,18,28^ and progenitor^18,26,27,42^ markers have been identified with unresolved connections. The discoveries of unipotent mammary stem cells for different mammary lineages under physiological condition further confounded the framework of mammary hierarchy^18,26,27,42^. Variable results have been generated in different labs with different mouse models, labeling efficiency, tracing schemes and analytical tools etc^18,28^. A central question is how different mammary populations in a unified condition fit into the same conceptual framework, and whether the identity of various functional populations can be pinpointed. In this study, we have systemically analyzed the spatial heterogeneity of mammary epithelial cells at the single-cell level. We isolated various mammary basal cell populations in the same mammary gland and characterized their distinct properties in colony formation, transplantation and lineage tracing assays. We demonstrated that both bipotent and unipotent mammary stem cells were present in the adult mammary gland but showed distinct kinetics in contributing to mammary homeostasis (Fig. 7t). This information helps to resolve the long-standing question that whether bipotent mammary stem cell exist in adult mammary gland, who they are and what they are doing. It will contribute to building the postnatal mammary hierarchy in the field.

Our discovery on the long-term contribution of bipotent mammary stem cells is crucial for us to understand the mammary physiology. First, these quiescent bipotent mammary stem cells are not easy to be efficiently labeled, and therefore could be easily missed in our biological and clinical studies. Second, since postnatal mammary gland is maintained by both bipotent and unipotent mammary stem cells, they could play different weights in various disease circumstances, especially for those chronic mammary complications. Third, bipotent mammary stem cells can still give rise to luminal cells at adult stage, thus it will be plausible that some luminal type of breast cancers, which is the majority of breast cancer in women, originating from bipotent basal cells. Consistent with this idea, activation of PIK3CA in basal cells indeed led to luminal type cancer in mouse^43^. Our genetic tracing tools will facilitate the future cancer of origin research for various types of breast cancers.

Our data supported a model that adult mammary gland is sustained by various stem cell populations including Bcl11b^+^ bipotent stem cells and Sema3a^+^ unipotent stem cells. However, these two stem cell populations may not be the only stem cells in the stem cell pool. In our tracing experiment, both Bcl11b^+^ and Sema3a^+^ cells are long-term survived stem cells; there should be at least short-term stem/progenitor cells or differentiated cells in the basal population that gradually diminish during the long-term tracing, because we clearly observed a significant decrease of basal coverage in Krt14 tracing experiment during the first pregnancy. The fate of these putative cell populations remains unclear and needs further investigated to find their markers. As to whether basal cell specification depends on Sema3a^+^ stem cells, we do not have data to support this idea. But from our data, we speculate that Sema3a^+^ cells are not the only unipotent mammary stem cells. The unipotent mammary stem cells might be a very heterogeneous population with distinct roles. In our current story, we are not able to conclude that. We hope in the future, we can comprehensively characterize the unipotent mammary stem cells population to reveal more mechanistic insights on mammary homeostasis.

Our study also has limitations. Our study was mainly conducted with mouse models, and it is still not clear whether it holds true in human breast tissues. The quiescent bipotent stem cells that we traced with *Bcl11b* mouse model were not complete for all the Bcl11b^+^ cells, therefore we might have missed those ‘deep quiescent’ cells in our tracing experiment. In addition, due to the lack of genetic mouse models, we were not able to trace every cell population of interest. For Bcl11b^+^ and Sema3a^+^ cells, due to the low frequency of these cells and low labeling efficiency, we were not able to efficiently detect their expression in scRNA-seq analysis, and not able to sort them out and directly conduct functional and characteristic analysis including their plasticity dynamics with aging. The mammary stem cell population might be very heterogenous, and there might be other bipotent and unipotent mammary stem cells that we did not have enough resources to cover. In the future, it will be interesting to further dissect the details of the lineage hierarchy, the inherent association between different cell populations and their significance in cancer initiation in both murine and human background.

## Materials and methods

### Ethics statement

All animal procedures reported herein were performed following the institutional guidelines and approved by the Institutional Animal Care and Use Committee at Westlake University (IACUC Protocol #19-001-CS).

### Mice

The *Bcl11b*^*flox/flox*^ mice (C57BL/6 background) were generously provided by Mark Leid’s lab, and were described previously^44^. The *TetO-Cre* (stock number 006234), *K14-rtTA* (stock number 007678), *mTmG* (stock number 007676), *Rosa26-tdTomato* (stock number 007909) mice were purchased from Jackson Laboratory. And the *Bcl11b-IRES-rtTA* (or *Bcl11b-rtTA*) mice were made by Shanghai Model Organisms Center, Inc. The frozen sperms of *Sema3a-CreERT2* mouse (stock number T000-620) were purchased from GemParmatech Co. Ltd., Nanjing, China. For analysis of pubertal mammary gland, 4–6-week-old female mice were used. For each experiment, the ages of the female mice used were indicated in the text. And all the mice used for the Bcl11b-lineage tracing assay had a genotype of *Bcl11b-rtTA (+, +) TetO-Cre (+) tdTomato (+, +)*. For the Sema3a-lineage tracing assay, the mice had a genotype of *Sema3a-CreERT2(+) tdTomato (+, +)*. And for the Krt14-lineage tracing assay, the mice had a genotype of *Krt14-rtTA (+, +) TetO-Cre (+) GFP (+, +)* with or without a homogeneous *Bcl11b (loxp, loxp)* background for *Bcl11b* deletion. All the mice were housed in specific pathogen-free conditions and bred in the laboratory animal resources center of Westlake University. Mice were administered with autoclaved food and water.

### Lineage tracing assay

For Bcl11b-lineage tracing experiments, 2 mg doxycycline (100 µL 20 mg/mL doxycycline in sterile PBS, Sangon Biotech) was injected intraperitoneally into the *TetO-Cre/Bcl11b-rtTA/tdTomato* mice once every day for three consecutive days starting at week 4. For the Krt14-lineage tracing experiments, a single dose of 2 mg doxycycline (20 mg/mL) was injected intraperitoneally into the *TetO-Cre/Krt14-rtTA/mTmG* mice at week 5. For the Sema3a-lineage tracing experiments, a single dose of 5 mg Tamoxifen (500 µL of 10 mg/mL tamoxifen in corn oil, Sigma) was injected intraperitoneally into the *Sema3a-CreERT2/tdTomato* mice at week 5. To evaluate the instantaneous labeling efficiency, the mammary glands were sampled 2 days after the last dose of induction and subjected to flow cytometry analysis. To quantify the tracing progenies during different stages of mammary gland development, the mice were sampled 3 weeks, 8 weeks, 20 weeks and 1 year after doxycycline/tamoxifen induction. To quantify the tracing outcomes during various stages of reproduction cycles, the drug-treated mice were mated in adulthood and then sampled on the 17.5^th^ day during the first, second, and third pregnancy. We also did complete involution between pregnancies, e.g., 1^st^ Involution means the involution between the first and second pregnancy for 30 days. Hereafter, ‘Invo2’ means involution for 2 days, and ‘Invo long’ indicates involution for more than 90 days. The other involutions between pregnancies unless specially stated were all 30 days. And ‘1^st^ Lac7’ indicates lactation for 7 days.

For lineage tracing clonal analysis, we dissected the individual bright tdTomato^+^ Bcl11b clones in the mammary fat pads during multiple pregnancies (1^st^, 2^nd^ and 3^rd^ Preg17.5 d) under the fluorescence stereomicroscope, and then digested them into single cells and performed the clonal analysis of the composition of basal and luminal lineage by FACS.

### Tissue processing and flow cytometry

All the mammary glands (1^st^, 2^nd^, 3^rd^, 4^th^ and 5^th^) from the pubertal (5−6 weeks), adult (>2 months) virgin or pregnant C57BL/6 mice were dissected and processed according to the published protocol^16^. Mammary glands were manually and mechanically minced into 1-mm size and digested with 0.5 mg/mL collagenase type 3 and 50 U/mL hyaluronidase (Stem Cell Technology) for 2 h with gentle pipetting every 30 min. Digested mammary homogenate was spun down at 1500 rpm in Thermo Centrifuge (Thermo Scientific), followed by 5 mL ACK lysing buffer (Beyotime) treatment for 5 min on ice to remove erythrocytes. Then, mammary cells were digested by 5 mL 0.25% Trypsin-EDTA (GIBCO) for 3−5 min, followed by brief DNase I (Worthington) digestion. Dissociated mammary cells were filtered by 40-μm strainer to obtain single cell suspension. For FACS analysis, mammary single cells were stained with CD45 (Biolegend), CD31 (Biolegend), Ter119 (Biolegend), CD49f (Biolegend), EpCAM (Biolegend), CD200 (Biolegend), CD34 (BD Bioscience), with appropriate conjugated fluorophores for 30 min on ice. Then cells were washed and resuspended in HBSS + 2%FBS + 1%P/S + DAPI (1 μg/mL) at a density of 10 million/mL. Stained samples were analyzed and sorted on BD FACSAria^TM^ Fusion (BD Bioscience) with a 100-μm nozzle.

To address the spatial heterogeneity of mammary gland, the nipple, duct and TEB fragments were dissected from the mammary glands of 5–6-week-old *Krt14-cre/mTmG* or wild-type (WT) C57BL/6 mice under the dissection microscope. The tissue fragments were then digested with collagenase type3 (0.5 mL, 3000 U/mL) at 37 ˚C for ~1.5 h until most of the large tissue pieces disappear. Wash tissues with HBSS (containing 1% P/S) at least twice. Add 300 μL TrypLE Express to digest the Nipples, Ducts and TEBs for 10 min. Add 1 mL HBSS (containing 2% FBS + 1% P/S) to neutralize TrypLE. Wash at least once with HBSS (containing 1% P/S). Resuspend cells in HBSS (containing 2% FBS + 1% P/S + DAPI (1 μg/mL)), count cells and adjust into 10 million/mL, and then stain the cells with appropriate combination of antibodies.

### Colony formation assay

To perform the colony formation assay, 30 μL of growth factor reduced Matrigel (BD Bioscience) was added into the 96-well round-bottom plate and then solidified at 37 ˚C for 10 min. 4 K/well CD200^−^CD34^+^, CD200^+^CD34^low^ and CD200^+^CD34^−^ basal cells were resuspended in 200 μL culture media (DMEM/F12 + 2% FBS + 1% P/S + B27 + 10 mM HEPES) supplemented with EGF (10 ng/mL, BD Bioscience), Rspo1 (250 ng/mL, R&D), ROCK inhibitor Y27632 (10 μM, Sigma), and were overlaid on top of the Matrigel. Plate was maintained in 37 ˚C incubator with 5% CO_2_ for 1−2 weeks.

### Transplantation assay

Transplantation assays were performed as previously described^16^. For this study, three subpopulations of pubertal basal cells (CD200^−^CD34^+^, CD200^+^CD34^low^ and CD200^+^CD34^−^) were sorted and resuspended in injection media (DMEM/F12 + 50% Matrigel + 1% P/S) to 50 K/5 μL, and serially diluted to 10 K/5 μL,1 K/5 μL, 200/5 μL 50/5 μL. Cells were injected into the cleared fat pad of 3-week-old recipient mice (C57BL/6) with designated dilutions. Seven weeks after the transplantation, the recipient mice were analyzed by mammary gland whole mount with carmine staining. The MRU frequency and confidence interval were determined by ELDA (http://bioinf.wehi.edu.au/software/elda/). As for the secondary transplantation, mammary outgrowths obtained from the first transplantation were harvested and processed into a single-cell suspension. Single-cell suspensions containing 4000 mammary cells from each mammary outgrowth were passaged to recipient mice for secondary transplantation.

### Real-time PCR

In total, 50–500 primary mammary cells or cultured epithelial cells of various populations were directly sorted into 400 μL Trizol (Life Technologies). RNA was extracted according to the manufacturer’s instruction with addition of ultrapure glycogen (Life Technologies) as carrier. RNA was reverse transcribed to cDNA using SuperScript III First Strand Synthesis Kit (Life Technologies) according to the manufacturer’s instructions. cDNA was preamplified 15–20 cycles according to the cell number using SybrGreen master mix (Applied Biosystems) and target gene primer pool designed by IDT (Integrated DNA Technologies). Preamplified cDNA was then subjected to the real-time PCR for specific gene target according to manufacturer’s instructions using Jena qTOWER384G. Data were analyzed by Graphad 8 and Excel. The data were normalized to the expression of β-actin. Then the value of one reference sample was set to 1 to facilitate inter-sample comparisons. Statistical analysis was performed using two-tailed unpaired *t*-test. Data were presented as means ± SEM, SYBR green primers are listed as below:

*β-Actin*: Forward ACCTTCTACAATGAGCTGCG, Reverse CTGGATGGCTACGTACATGG;

*Bcl11b*: Forward AGGAGAGTATCTGAGCCAGTG, Reverse GTTGTGCAAATGTAGCTGGAAG;

*Tbx2*: Forward CCTTCCGCACCTATGTCTTC, Reverse TGCTTCCTTTTCTCCCGAC;

*Tspan8*: Forward AGTTCCGTTTACCCAAAGACC, Reverse GCACCATAGAAAACACCAAACC;

*Mycn*: Forward GTCTGTTCCAGCTACTGCC, Reverse TCCTCTTCATCTTCCTCCTCG；

*Sema3a*: Forward TCGGCAATGGAGCTTTCTAC, Reverse CCATCCCAGGCACAGTAAG;

*Actg2*: Forward CCTAAACCCCAAAGCAAACAG, Reverse CCCCGAATCCAGAACGATG;

*Sfrp1*: Forward CTCTAAGCCCCAAGGTACAAC, Reverse TCTTGTCACCGTTTTCCTTCT;

*Slc6a15*: Forward CCGACACATGTTCACTCCTAAG, Reverse CACCAAATCCCAAACCCAATG;

*Csn3*: Forward GCAGAGATACAAAACCCAGATTC, Reverse GTACAGGACTCTTTGCTCATCG; *Igfbp2*: Forward GAACATCTCTACTCCCTGCAC, Reverse TCCGTTCAGAGACATCTTGC;

*Alpl*: Forward CTCCAAAAGCTCAACACCAATG, Reverse ATTTGTCCATCTCCAGCCG;

*Ccdc129*: Forward CATCTGCTTCCTGACTCTGAG, Reverse GTATGGTTGATGGTTTGCTGG;

*Slpi*: Forward TGAATCCTGTTCCCATTCGC, Reverse ACATATACCCTCACAGCACTTG;

*Lgr5*: Forward CACCCCAATGCGTTTTCTAC, Reverse GATGGTATCAGGCTCTGTAAGG;

*Cyp1b1*: Forward CCACTATTACGGACATCTTCGG, Reverse CACAACCTGGTCCAACTCAG;

*Ttc9*: Forward TGAAGCCATTGAGATCGACTG, Reverse CCACACCAGACCTGTAAAGAG;

*Ereg*: Forward CTGCCTCTTGGGTCTTGACG, Reverse GCGGTACAGTTATCCTCGGATTC.

**Paraffin sections and immunofluorescence** For paraffin sections, mammary gland was dissected and immediately fixed by 4% PFA for 2 h at 4 ˚C followed by PBS washing. Dehydrated by gradient ethanol (75%, 80%, 95%,100%) solution. Dehydrated tissue was infiltrated by xylene solution and embedded with paraffin. Tissue block was sectioned to 5 μm using Rotary Microtome Leica RM2255 (LEICA). To do the immunofluorescence, paraffin section was de-paraffinized using xylene and rehydrated with gradient (100%, 95%, 80%, 75%, 0%) ethanol solution and subjected to immunofluorescence staining. Antigen was retrieved in citrate buffer (10 mM Sodium Citrate, 0.05% Tween 20, pH 6.0) boiled for 15 min in microwave at low-to-medium power. Then sections were blocked with TBS + 2% BSA + 5% Donkey serum + 0.1%Triton X-100 for 1 h at RT, and then stained with primary antibody including Rat-anti CD34(BD Biosciences), Rat anti-Bcl11b (Abcam), Rabbit anti-Krt14 (Biolegend), Rabbit anti-Mycn (Cell Signaling Technology), monoclonal anti-αSMA (Sigma), Goat anti-RFP (biorbyt) overnight at 4 ˚C, followed by 3× washing by TBST and secondary antibody staining Donkey anti-Rat, Rabbit, Mouse or Goat 1:200 (Jackson ImmunoResearch) at RT for 1 h. After 3× TBST washing and brief DAPI staining (1 μg/mL), sections were mounted with Fluoromount Aqueous Mounting Medium (Sigma). Seal coverslips with nail polish to prevent drying and movement under microscope. Store in dark at −20 °C.

### 3D tissue clearing, imaging and analysis

The mammary glands were dissected and immediately fixed by 4% PFA at 4 ˚C overnight, 360° shaking. Then wash with 0.1%TBST (0.1% Triton X-100 in PBS) 3× 1 h, angle-shaking. Then stain DAPI (1 μg/mL) overnight in 1% TBST, angle-shaking. Then wash with TBST 3× for 1 h, 4 ˚C, angle-shaking. Block with 3% NCS (new born calf serum) in 1% TBST overnight 4 ˚C, angle-shaking. Stained with primary antibody in 1% TBST + 3% NCS + 0.03% NaN3 overnight at 4 ˚C, angle-shaking. Then move to 37 ˚C, horizontally shaking (450 pm) for 5 days. Then wash with 0.1% TBST 3× 1 h, 4 ˚C, angle-shaking. Dehydrated with gradient (25%, 50%, 75%, 100%) methanol solution RT, 360° shaking. Then treat with DCM overnight 4 ˚C, angle shaking, to let tissue descend to the bottom. Stain with DAPI (1 μg/mL) in DBE, angle-shaking overnight if necessary. Then transfer the tissue to fresh DBE, 4 ˚C, angle-shaking. Tissue can be kept in DBE. Then take pictures of the tissue with a confocal microscope (FV3000, Olympus) compatible with DBE. Data were processed and analyzed by Imaris software version 9.6.

### Clonal analysis

We define tdTomato-positive clone as a cluster of cells with bright tdTomato fluorescence with a clear boundary from the peripheral cells. To count the number of clones formed during Bcl11b- and Sema3a-lineage tracing, the mammary gland is regarded as an ellipse, and its longest axis is divided into three equal parts, resulting in three regions. The region closest to the nipple is called the proximal region, the region farthest from the nipple is called the distal region, and the region between them is called the middle region. The areas of the three regions are denoted as S1, S2, and S3, respectively. After initially counting the number of clones in each region, the clone counts are normalized based on the different areas of the three regions.

In terms of the specific methods for clone type analysis, we employed two approaches. The first approach involves directly using ophthalmic microsurgical scissors under a fluorescence stereo microscope to collect larger, visibly tdTomato-positive clones from the mammary glands of pregnant mice or mice at other indicated time points after pubertal pulsing, while minimizing the collection of surrounding tissue. Each clone is then placed in a separate 1.5-mL centrifuge tube and digested into a single-cell suspension using enzymatic digestion. The clone composition of basal and luminal lineage is analyzed using flow cytometry with relevant antibodies. The second method is to analyze the composition of each individual clone regardless of size by performing immunofluorescence staining of basal and luminal markers for mammary sections of pregnant mice or virgin mice at designated time points after pubertal pulsing. Under the microscope, we observe and quantify the composition of basal and luminal cells in different tdTomato-positive clones in mice.

### scRNA-seq

Single cells dissociated from nipples, ducts and TEBs of mouse mammary gland were sorted by FACS (MoFlo Astrios, Beckman Coulter) into individual well of 96-well PCR plates containing preloaded lysis buffer ERCC spike-in and barcode. Libraries were constructed as previously described^45^. To lyse the cells, the 96-well plate was first incubated in 72 ˚C for 3 min and then transferred to ice immediately. 2.85 μL of RT mixture containing 40 U SuperScript II reverse transcriptase (Invitrogen), 5U RNase Inhibitor (Takara), 5× Superscript II first-strand buffer, 25 mM dithiothreitol, 5 M betaine (Sigma-Aldrich), 30 mM MgCl2 (Sigma-Aldrich), and 1.75 μM template switch oligo (TSO) primer was added into the lysate. The reverse transcription was conducted at 25 ˚C for 5 min, 42 ˚C for 60 min, 50 ˚C for 30 min, and then 70 ˚C for 10 min. Next, 7.5 μL of PCR mixture containing 6.25 μL 2× KAPA HiFi HotStart ReadyMix (KK2602), 300 nM ISPCR oligo (AAGCAGTGGTATCAACGCAGAGT) and 1 μM 3’ Anchored oligo (GTGACTGGAGTTCAGACGTGTGCTCTTCCGATC) were added to each reaction. The sample was amplified with initial denaturation at 95 ˚C for 3 min, then 4 cycles of 98 ˚C for 20 s, 65 ˚C for 30 s, and 72 ˚C for 5 min, followed by 10–16 cycles of 98 ˚C for 20 s, 67 ˚C for 15 s, and 72 ˚C for 5 min; and finally 72 ˚C for 5 min. Then the PCR products with different barcodes were pooled together and purified with DNA Clean & Concentrator-5 once (Zymo Research), eluted in 50 μL of H_2_O following 0.8× XP beads (Beckman, A63881 AMPure XP) purification twice, finally eluted in 21 μL H_2_O. Next, the cDNAs were amplified with biotinylated index primer (/Biotin/CAAGCAGAAGACGGCATACGAGATindexGTGACTGGAGTTCAGACGTGTGCTCTTCCGATC) and ISPCR oligo for an additional four to five cycles following purification with 0.8× Ampure XP beads again. The biotinylated cDNAs were sonicated (COVARIS #SIAUH006) into ~300-bp fragments. To enrich the amplified products, Dynabeads MyOne Streptavidin C1 Beads (Thermo Fisher Scientific) were used following manufacturer’s instructions. Libraries were prepared using KAPA Hyper Prep Kits (KK8505) with end repair, A-tailing, and adapter ligation by using NEB U-shaped adapter. After post-ligation cleanup, libraries were amplified 7−8 cycles followed by purification with 0.8x Ampure XP beads twice and eluted in 30 μL of H_2_O. Quality was checked by Fragment Analyzer-12/96 (GENE-QC006). Finally, the libraries were sequenced on a Novaseq platform to generate 150-bp paired-end reads (sequenced by Novogene).

### scRNA-seq data analysis

Adaptors of raw reads were trimmed by TrimGalore. Low-quality reads were removed by FastQC. The information of cell barcode and UMI were recognized and extracted from reads1 by using UMI-tools and added to reads2. Those reads2 were mapped to reference genome, mm10, by STAR with default parameter except for outFilterMultimapNmax = 1. Then we used featureCount to assign mapped reads and used UMI-tools to produce a count matrix. After obtaining the count matrix, we did all downstream analyses with R. We kept the cells whose barcodes exist in the barcode library. We got the final dataset containing 1419 cells with a median of 36,842 UMIs and 3198 genes. We used ERCC as an external RNA control, so we filtered out the cells with ERCC of more than 5%. We also filtered out the poor quality cells with UMI numbers higher than 120,000 or less than 5000, and unique gene counts over 6000 or less than 200. R package Seurat v3.6 was used to normalize and scale data, and downstream including dimensionality reduction, clustering, tSNE plot, and so on. We regressed out the ERCC percentage effect, cell cycle effect, and gene number effect when scaling data. We used the top 2000 high variable features to do dimensionality reduction and used the first 14 principles components to run RunTSNE. We used the Wilcox as testing method. We used R package clusterProfiler to do the pathway enrichment analysis. We enriched the significantly changed pathway with FDR < 0.05. The expression of some marker genes was plotted by Seurat or ggplot2 R package.

### Bcl11b activity analysis

We used Bcl11b targets from our lab’s previous study^46^ to compute its activity. Briefly, we did ChIP-seq assay to find out Bcl11b target genes. Basal on scRNA-seq data of CD49f^high^ EpCAM^low^ Lin^−^ cells from WT-Basal and *Bcl11b*-KO C57BL/6 mice, we got Bcl11b positively regulated genes (genes with *P* value < 0.05 and KO < WT intersecting with Bcl11b targets) and negatively regulated genes (genes with *P* value < 0.05 and KO > WT intersecting with Bcl11b targets). Bcl11b activity was computed as weighted average expression of the negatively regulated genes. As to the regulon, we have tried to perform this analysis using our single-cell data. Unfortunately, the cell number and the expression level of Bcl11b do not support a robust Bcl11b regulon analysis. To overcome this limitation, we utilized an embryonic dataset from Cedric Blanpain’s published paper on NCB^47^, where Bcl11b is highly expressed. Using SCENIC^48^, we successfully identified the Bcl11b regulon regulated by the Bcl11b’s transfac_pro__M05956 motif. By computing the activity of this regulon in our dataset, we observed results consistent with our previous computational methods.

### Data analysis and related softwares

Graphad Prism 9 was used for statistical analysis. Data were compared between two groups of samples using the unpaired, two-tailed Student’s *t-*test. Sample size for each experiment was determined based on a minimum of *n* = 3 independent devices for each experimental group. All data were presented as means ± SEM. *P < 0.05, **P < 0.01, ***P < 0.001. Snapgene was used for primer design. Flowjo 10 was used for FACS data analysis. Image J and Photoshop were used for image analysis. And Adobe Illustrator CS6 was used for image layout and editing.

## Supplementary information

Supplementary Figures

## Data Availability

The data that support the findings of this study are available from the corresponding author upon reasonable request. Source data are provided in the manuscript. Singel-cell data were deposited in GEO with the access number GSE251933.
