# Supplementary material for: Distinct mammary stem cells orchestrate long-term homeostasis of adult mammary gland
Source: Cell Discov. 2025 Apr 15;11:39. doi: 10.1038/s41421-025-00794-0 (PMC12000503; doi:10.1038/s41421-025-00794-0)
Supplement: Supplementary file 1 — Supplementary Figures [file 41421_2025_794_MOESM1_ESM.pdf]

Supplementary Fig. 1

a

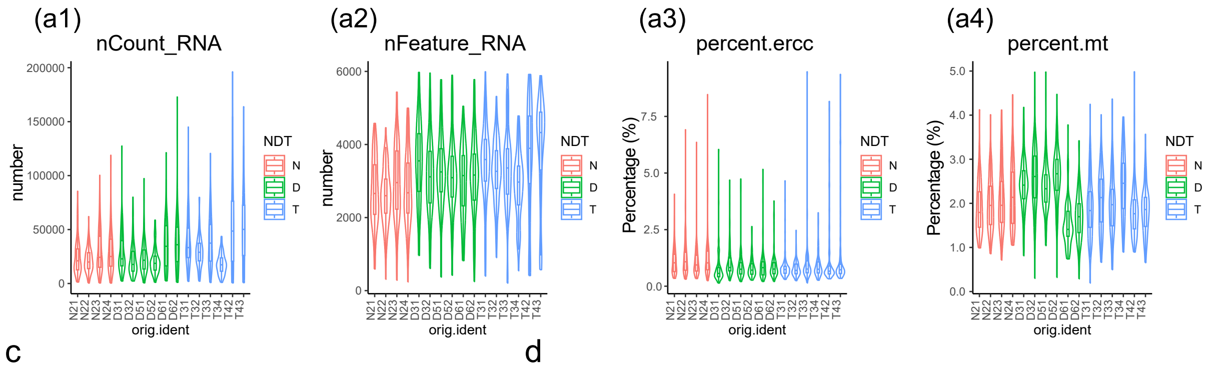

b

Sample	Cell number
N21	86
N22	92
N23	91
N24	90
D31	84
D32	91
D51	89
D52	89
D61	83
D62	87
T31	90
T32	86
T33	89
T34	84
T42	81
T43	85

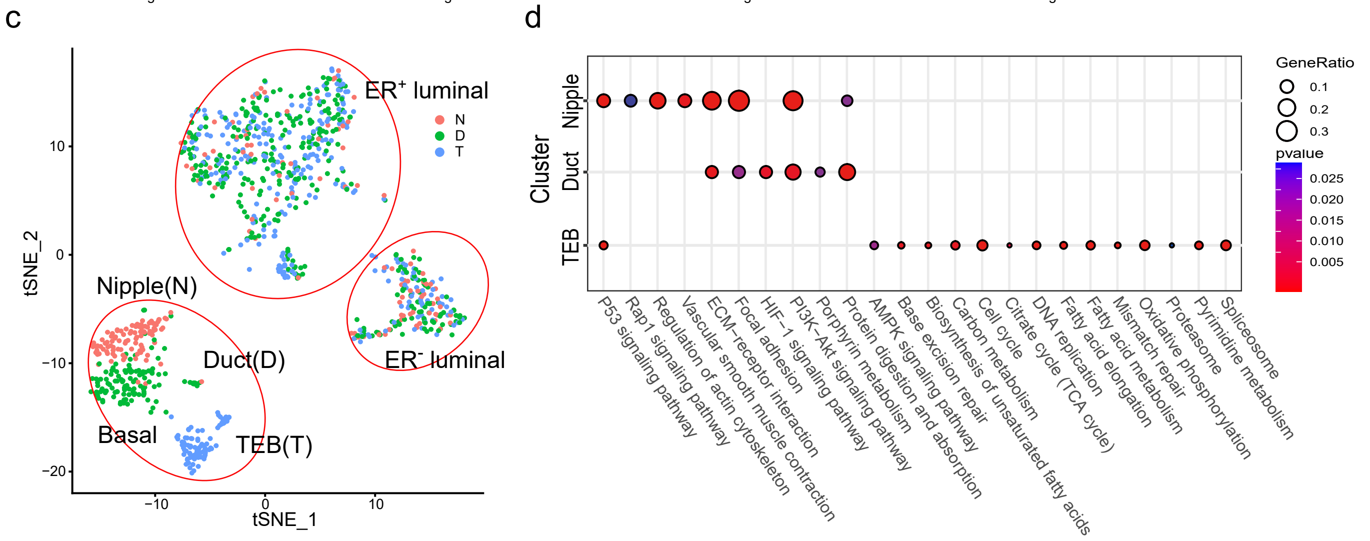

Supplementary Fig. 1 ScRNA-seq clustering and pathway analysis under stringent quality control for cells in different locations of pubertal (5w) mouse mammary, including TEBs, ducts, and nipples. (**a**) ScRNA-seq quality control. (a1) Number of Unique Molecular Identifiers (UMIs) detected per cell, a measure of sequencing depth. (a2) Number of genes detected per cell, reflecting the complexity of the transcriptome. (a3) Distribution of the proportion of ERCC (External RNA Controls Consortium) spike-in transcripts for each sample. (a4) Distribution of the proportion of reads mapped to genes in the mitochondrial genome for each sample. **(b**) Number of cells per sample in scRNA-seq analysis. All samples were dissected from 5-week-old *Krt14-Cre*/*Rosa26-mTmG* mice with C57BL/6 background. N for nipple sample, D for duct sample, T for TEB sample. All samples were dissected from 5-week-old *Krt14-Cre*/*Rosa26-mTmG* mice with C57BL/6 background. (**c**) t-SNE plots showing the clustering of mammary epithelial cells from various locations. (**d**) KEGG pathway analysis for basal cells in TEB, duct and nipple.

Supplementary Fig. 2

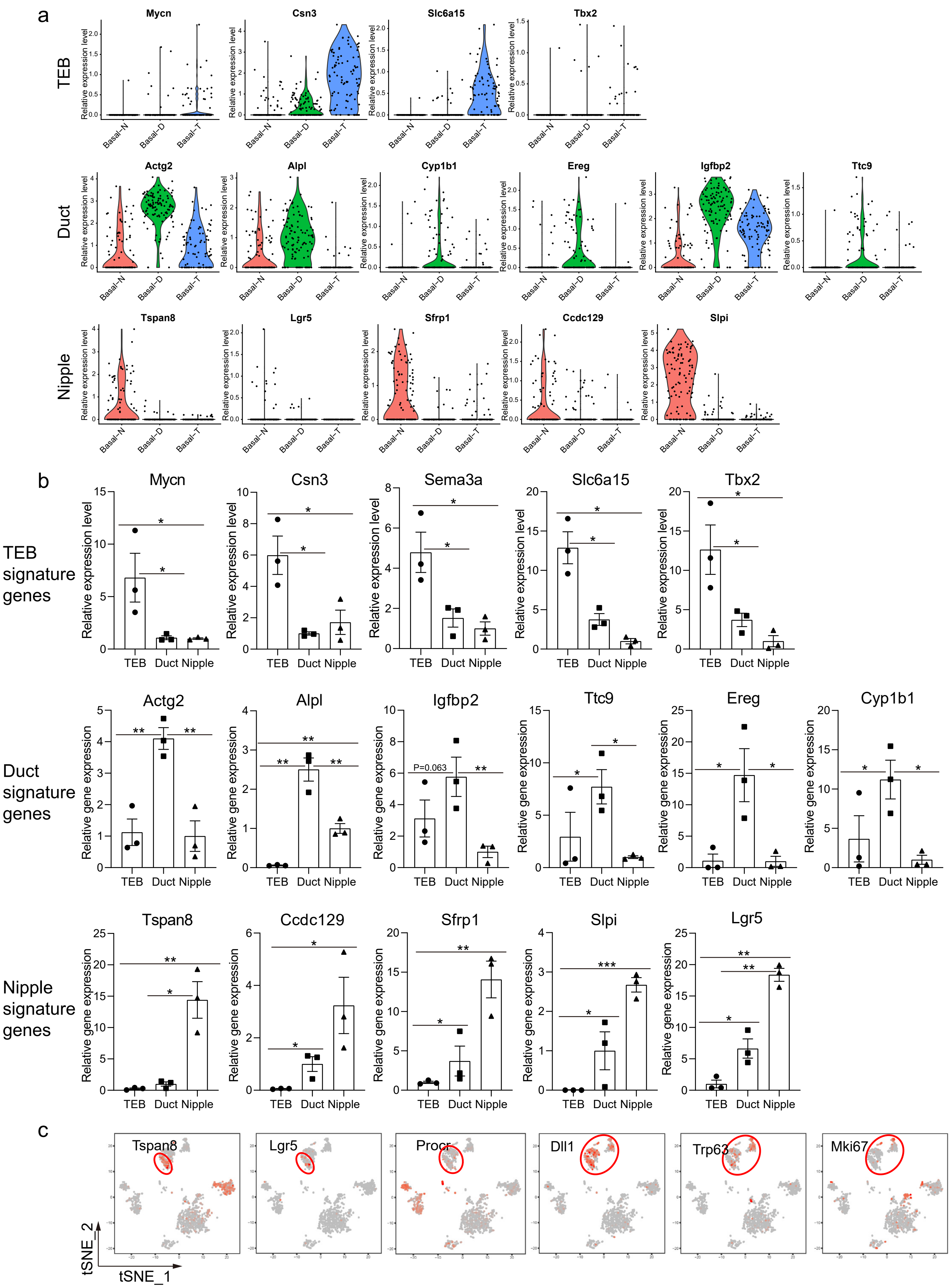

Supplementary Fig. 2 Heterogeneity for mammary epithelial cells. (**a**) Violin plots showing signature genes for basal cells in TEB, duct and nipple in scRNA-seq analysis. (**b**) Real-time PCR analysis showing the relative expression level of signature genes in basal cells in TEB, duct and nipple, exhibiting expression patterns consistent with those illustrated in (**a**). All the data were normalized to the expression of *β-actin*. Statistical analysis was performed using two-tailed unpaired t-test. Data were presented as mean ± SEM, n = 3. *p<0.05, **p<0.01, ***p<0.001. (**c**). tSNE plots showing the reported signature genes for mammary stem and progenitor cells in scRNA-seq analysis.

Supplementary Fig. 3

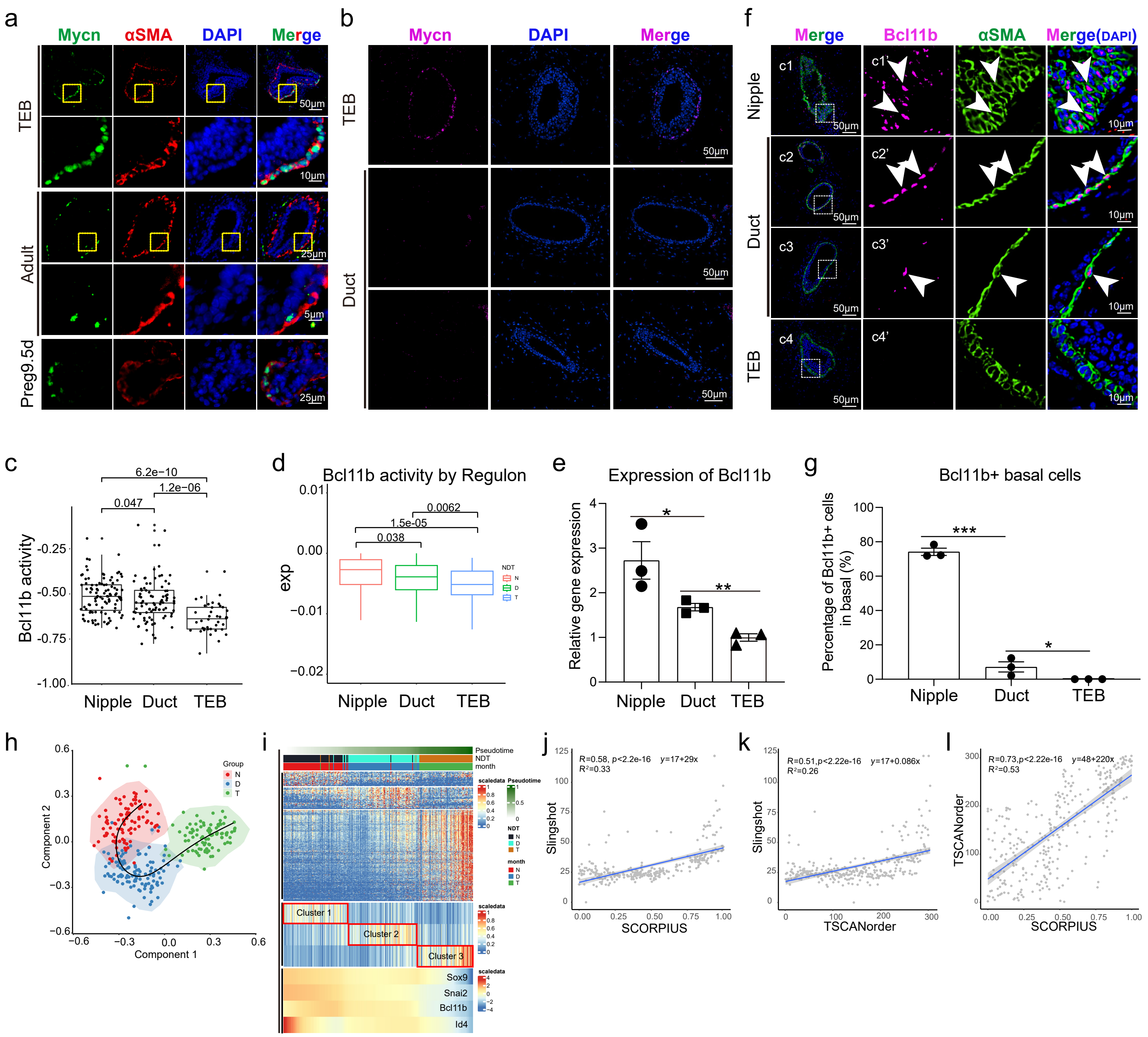

Supplementary Fig. 3 Signature gene verification and trajectory analysis of basal cells in different locations of nipple, duct and TEB. (**a-b**) Representative immunofluorescence imaging of the expression of Mycn in TEB, duct and alveolar basal cells. Magenta/GFP: Mycn; Red: αSMA; blue: DAPI. Scale bar, 5μm, 10μm, 25μm or 50μm. (**c**) Bcl11b activity determined by Bcl11b ChIP targets in basal cells from nipple, duct and TEB. (**d**) Bcl11b activity by regulon analysis. (**e**) Real-time PCR quantification of *Bcl11b* expression in basal cells from nipple, duct and TEB. The data was normalized to the expression of *β-actin*. Statistical analysis was performed using two-tailed unpaired t-test. Data were presented as mean ± SEM, n = 3. $^*p<0.05$, $^{**}p<0.01$.(**f**) Representative immunofluorescence images of the expression of Bcl11b in nipple, duct and TEB. The areas enclosed by the dashed line in the images were enlarged on the right. The white arrows indicate $Bcl11b^+$ basal epithelial cells. Magenta, Bcl11b; green, αSMA; blue, DAPI. Scale bar, 10μm or 50μm. (**g**) Quantification of the percentage of $Bcl11b^+$ basal cells in (**f**). Statistical analysis was performed using two-tailed unpaired t-test. Data were presented as mean ± SEM, n = 3. $^*p<0.05$, $^{***}p<0.001$. (**h**) Trajectory analysis of pubertal basal cells across nipple, duct, and TEB using the SCORPIUS R package. (**i**) Differential gene expression analysis along pseudotime trajectory defines three gene sets corresponding to nipple, duct, and TEB, with higher activity of canonical stem cell markers in nipple and lowest in TEB. (**j-l**) Comparison of pseudotime inference using three trajectory inference methods including Slingshort, SCORPIUS and TSCANorder.

Supplementary Fig. 4

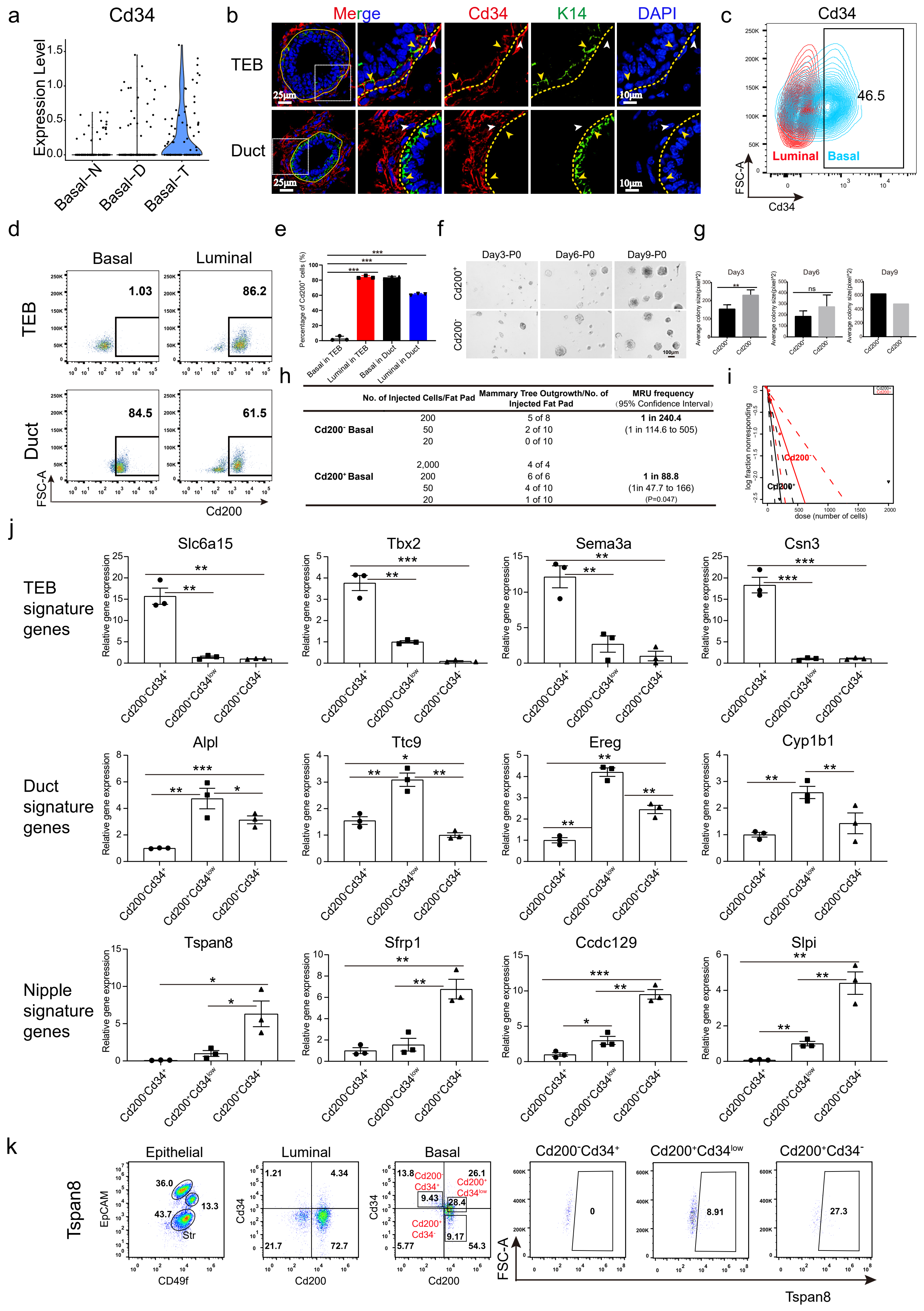

	No. of Injected Cells/Fat Pad	Mammary Tree Outgrowth/No. of Injected Fat Pad	MRU frequency (95% Confidence Interval)
Cd200⁻ Basal	200<br>50<br>20	5 of 8<br>2 of 10<br>0 of 10	**1 in 240.4**<br>(1 in 114.6 to 505)
Cd200⁺ Basal	2,000<br>200<br>50<br>20	4 of 4<br>6 of 6<br>4 of 10<br>1 of 10	**1 in 88.8**<br>(1in 47.7 to 166)<br>(P=0.047)

Supplementary Fig. 4 Cd34 and Cd200 separates basal cells into distinct subpopulations. (**a**) Violin plot showing the single cell expression level of Cd34 in basal cells from TEB, duct and nipple. (**b**) Representative immunofluorescence images showing the expression of Cd34 in TEB and duct from a 6-week-old mouse. The yellow arrows indicate basal cells, and the white arrows indicate stromal cells. Red, Cd34; green, Krt14; blue, DAPI. Scale bar, 10μm or 25μm. (**c**) Representative FACS plot showing the expression of surface marker Cd34 in basal and luminal cells from mammary glands of a 6-week-old mouse. (**d**) Representative FACS plots showing the expression of Cd200 in basal and luminal cells from TEB and duct respectively. (**e**) Bar chart showing the percentage of $Cd200^+$ cells in basal and luminal cells from TEB and duct respectively. Statistical analysis was performed using two-tailed unpaired t-test. Data were presented as mean ± SEM, n = 3. ***$p<0.001$. (**f**) Colony formation assay for $Cd200^+$ and $Cd200^-$ primary basal populations isolated from mammary glands of 6-week-old mice. Four thousand cells per well were seeded for each condition and cultured for 2 weeks. Scale bar, 200μm. (**g**) Bar chart showing the average colony sizes for the cultured cells on Day3, Day6 and Day9. Statistical analysis was performed using two-tailed unpaired t-test. Data were presented as mean ± SEM, n = 3. **$p<0.01$, ns, not significant. (**h**) Data table showing the numbers of total injections, outgrowths, and repopulating frequency of $Cd200^+$ and $Cd200^-$ basal populations isolated from mammary glands of 6-week-old pubertal mice in the cleared fat pad transplantation assay. (**i**) ELDA plot of limiting dilution transplant showing the frequency of repopulating unit in the two different subpopulations of $Cd200^+$ and $Cd200^-$ basal cells. (**j**) Real-time PCR analysis showing the relative expression level

of signature genes in different basal subpopulations divided by Cd200 and Cd34 in pubertal mammary glands of 6-week-old mice. The data were normalized to the expression of *β-actin*. Statistical analysis was performed using two-tailed unpaired t-test. Data were presented as mean ± SEM, n = 3. *p<0.05, **p<0.01, ***p<0.001. (**k**) Representative FACS plots showing the distinct expression level of Tspan8 in three different basal subpopulations divided by Cd200 and Cd34.

Supplementary Fig. 5

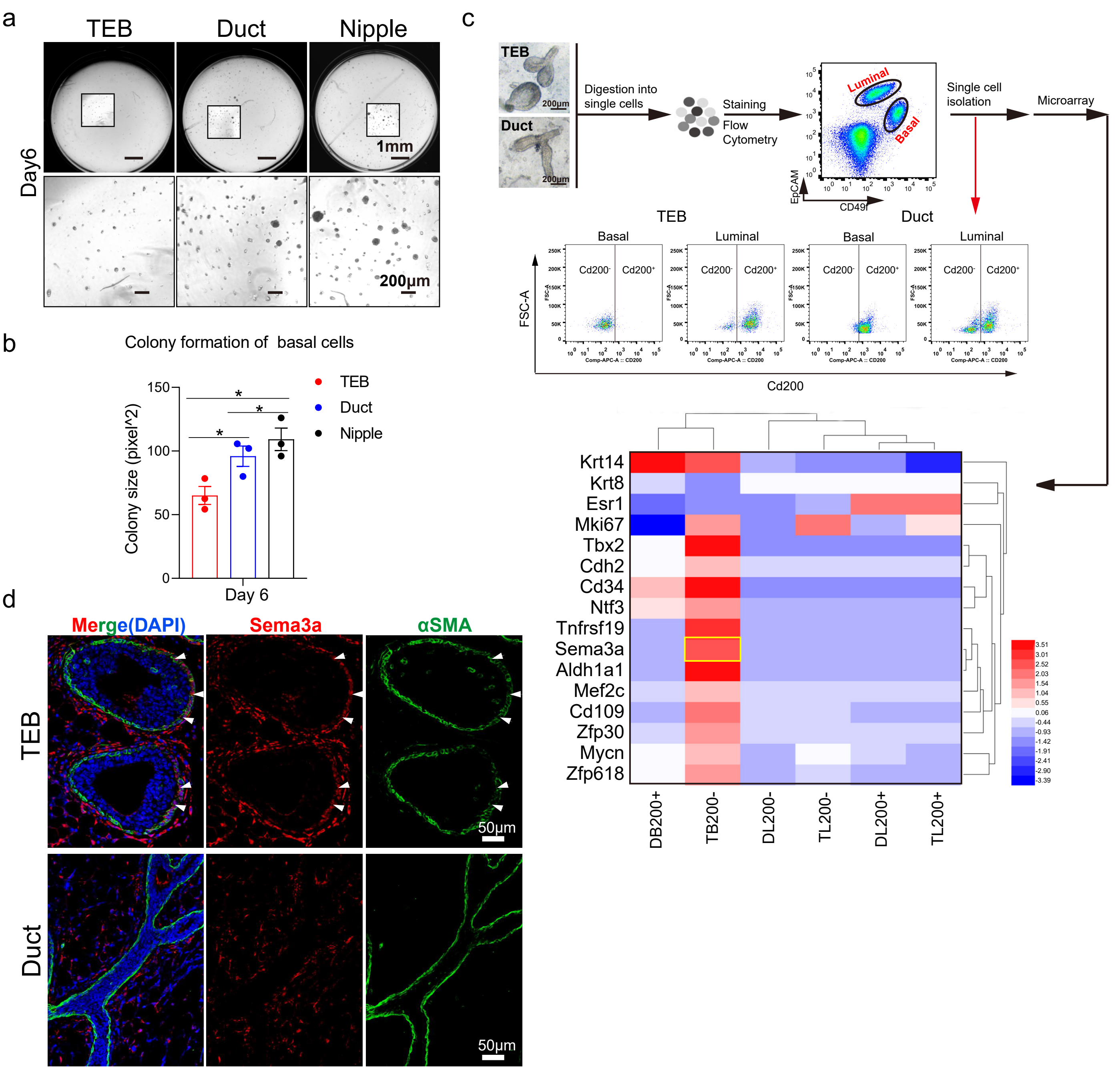

Supplementary Fig. 5 Distinct clonal formation characteristics and molecular features of basal cells in different locations across TEB, duct and nipple. (**a**) Representative images of colony formation assay of different basal cells in TEB, duct and nipple from 3 replicates on day 6 are shown. Scale bar, 200μm or 1mm. Three thousand basal cells were seeded into each well. The regions selected by the frame in the upper panel were enlarged in the lower panel. Scale bar, 200μm or 1mm. (**b**) Colony sizes were measured and plotted in the bar graph. Statistical analysis was performed using two-tailed unpaired t-test. Data was presented as mean±SEM, n=3, *p<0.05. (**c**) Isolation of $Cd200^+$ and $Cd200^-$ basal and luminal populations by FACS and then performing microarray analysis. Mammary tissue structures including TEBs and ducts were dissected from 5- to 6-week-old pubertal C57BL/6 mice and digested into single cells, and flow cytometry (FACS) was used to isolate distinct basal and luminal cell populations based on the surface marker Cd200. Subsequently, these isolated populations underwent microarray analysis. Heatmap displaying the expression profiles of characteristic genes specific to basal and luminal populations by Cd200 in pubertal mouse mammary. DB200+: $Cd200^+$ basal cells in duct; TB200-: $Cd200^-$ basal cells in TEB; DL200-: $Cd200^-$ luminal cells in duct; TL200-: $Cd200^-$ luminal cells in TEB; DL200+: $Cd200^+$ luminal cells in duct; TL200+: $Cd200^+$ luminal cells in TEB. (**d**) Representative IHF co-staining of Sema3a and basal marker αSMA in TEB (upper) and duct (lower) of 5- week-old mice are shown. Basal cells at the tip of TEB and stromal cells surrounding glands are positive for Sema3a staining (the white arrows indicate $Sema3a^+$ basal cells). Red: Sema3a; green: aSMA; blue: DAPI. Scale bar, 50μm.

Supplementary Fig. 6

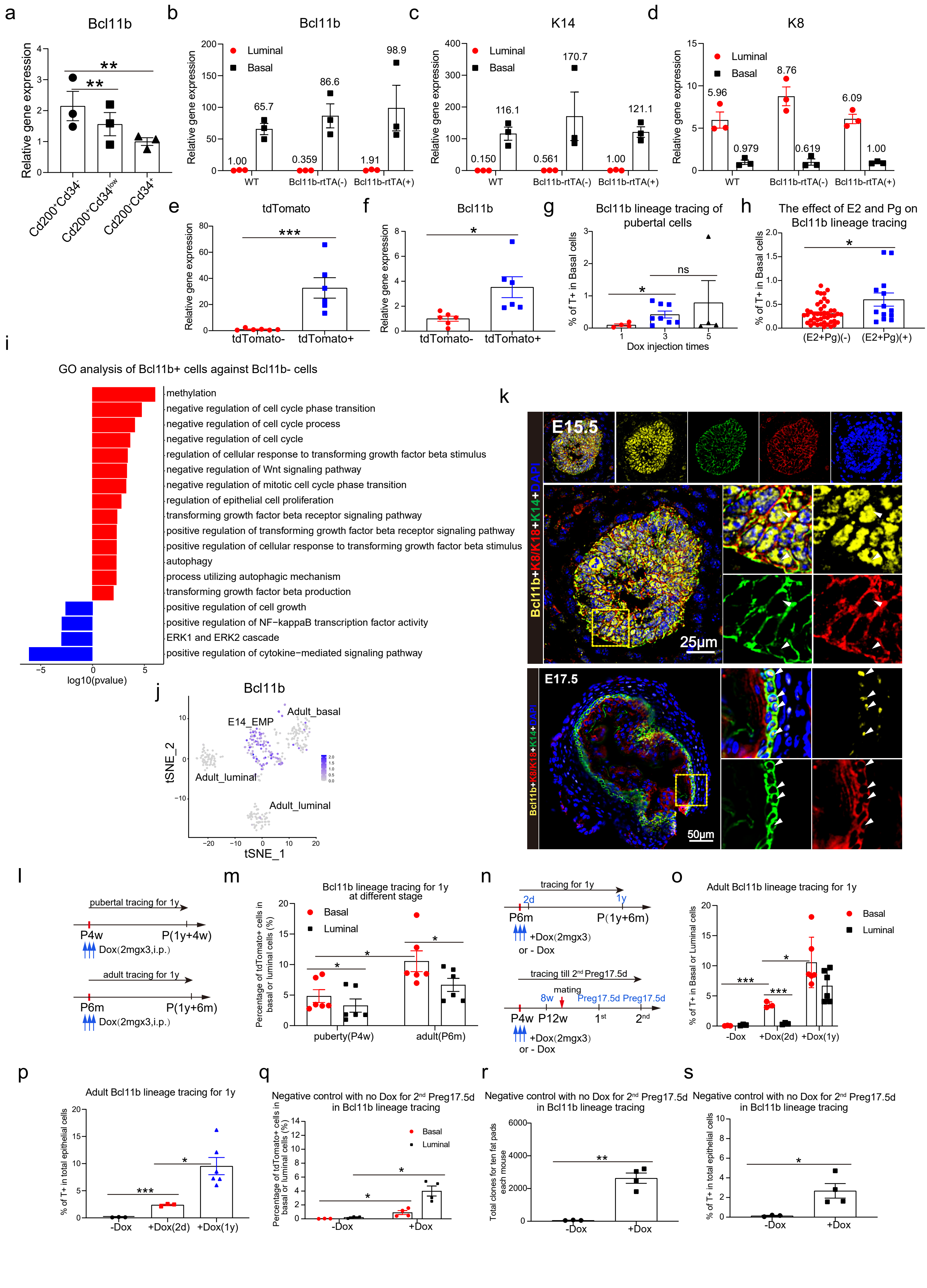

Supplementary Fig. 6 Low labeling efficiency of Bcl11b$^+$ cells and adult Bcl11b tracing with negative control. (**a**) Real-time PCR analysis showing the relative expression level of *Bcl11b* in different basal subpopulations divided by Cd200 and Cd34. The data were normalized to the expression of *β-actin*. Statistical analysis was performed using two-tailed unpaired t-test. Data were presented as mean ± SEM, n = 3. **p<0.01. (**b-d**) Stable expression of Bcl11b indicates no effect from insertion of IRES-rtTA cassette**.** *Bcl11b-IRES-rtTA/TetOCre/tdTomato* mice at 5 weeks old were treated with or without Dox (3x2mg/mouse), and the mammary glands were harvested 2 days later. The basal and luminal cells of each group of mice were separated by FACS and then qPCR quantification analysis was performed to detect the expression level of Bcl11b (**b**), Krt14 (**c**) and Krt8 (**d**) relative to that of *β-Actin*. Data was presented as Mean±SEM, n=3. (**e-f**) Real-time PCR analysis showing the relative expression level of *tdTomato* (**e**) and *Bcl11b* (**f**) in tdTomato-labeled and unlabeled basal cells 2 days after pubertal Bcl11b tracing. The data were normalized to the expression of *β-actin*. Statistical analysis was performed using two-tailed unpaired t-test. Data were presented as mean ± SEM, n=6. *p<0.05, ***p<0.001.(**g**) Bar chart showing the induction efficiencies with different times of Dox (2mg each time per day). Statistical analysis was performed using two-tailed unpaired t-test. Data were presented as mean ± SEM, n = 4-8. *p<0.05, ns, not significant. (**h**) Bar chart showing the percentage of tdTomato-labeled basal cells for 1mg 17β-Estradiol (E2) and 10µg progesterone (Pg)-treated mice. Statistical analysis was performed using two-tailed unpaired t-test. Data were presented as mean ± SEM, n = 11-45. *p<0.05. (**i**) Gene Ontology (GO) pathway analysis revealed the upregulated (red) and downregulated (blue) signaling pathways enriched in

$Bcl11b^+$ basal cells against $Bcl11b^-$ basal cells. The data of adult mammary epithelial cells for GO analysis originates from the scRNA-Seq data in the Tabula Muris database (https://tabula-muris.ds.czbiohub.org/). (**j**) ScRNA-Seq analysis of the data of both embryonic and adult mouse mammary epithelial cells from the paper by Cedric Blanpain in 2018 (https://doi.org/10.1038/s41556-018-0095-2). (**k**) Representative immunofluorescence images showing the expression of Bcl11b in specific mammary epithelial cells at E15.5 (the upper panel) and E17.5 (the lower panel**)**. The selected regions by the yellow boxes were split for different channels on the right. The white arrows indicate the $Bcl11b^+$ epithelial (basal) cells. Yellow, Bcl11b; red, Krt8/Krt18; green, Krt14; blue, DAPI. Scale bar, 25μm or 50μm. (**l**) Schematic diagram showing the tracing strategy of $Bcl11b^+$ cells during puberty and adult stage. (**m**) Bar chart showing the percentage of tdTomato-labeled cells in basal or luminal populations at the indicated times during puberty (4-week-old) and adult (6-month-old) in Bcl11b-lineage tracing. Statistical analysis was performed using two-tailed unpaired t-test. Data were presented as mean ± SEM, n = 6. *$p<0.05$. (**n**) Schematic diagram showing the tracing strategy of $Bcl11b^+$ cells during puberty (4-week-old) and adult (6-month-old) stage with or without Dox treatment. (**o-p**) Bar charts showing the percentage of tdTomato-labeled cells in basal or luminal populations (**o**), or the percentage of tdTomato-labeled epithelial cells (**p**) after adult tracing at 6 months for 1 year.(**q-s**) Bar charts showing the percentage of tdTomato-labeled cells in basal or luminal populations (**q**), the number of total clones for each mouse (**r**), or the percentage of tdTomato-labeled epithelial cells (**s**) at the 2nd Preg17.5 after pubertal tracing at 4 weeks with or without Dox treatment. All above statistical analysis was performed using two-tailed

unpaired t-test. Data were presented as mean ± SEM, n = 3-4. *p<0.05, **p<0.01.

Supplementary Fig. 7

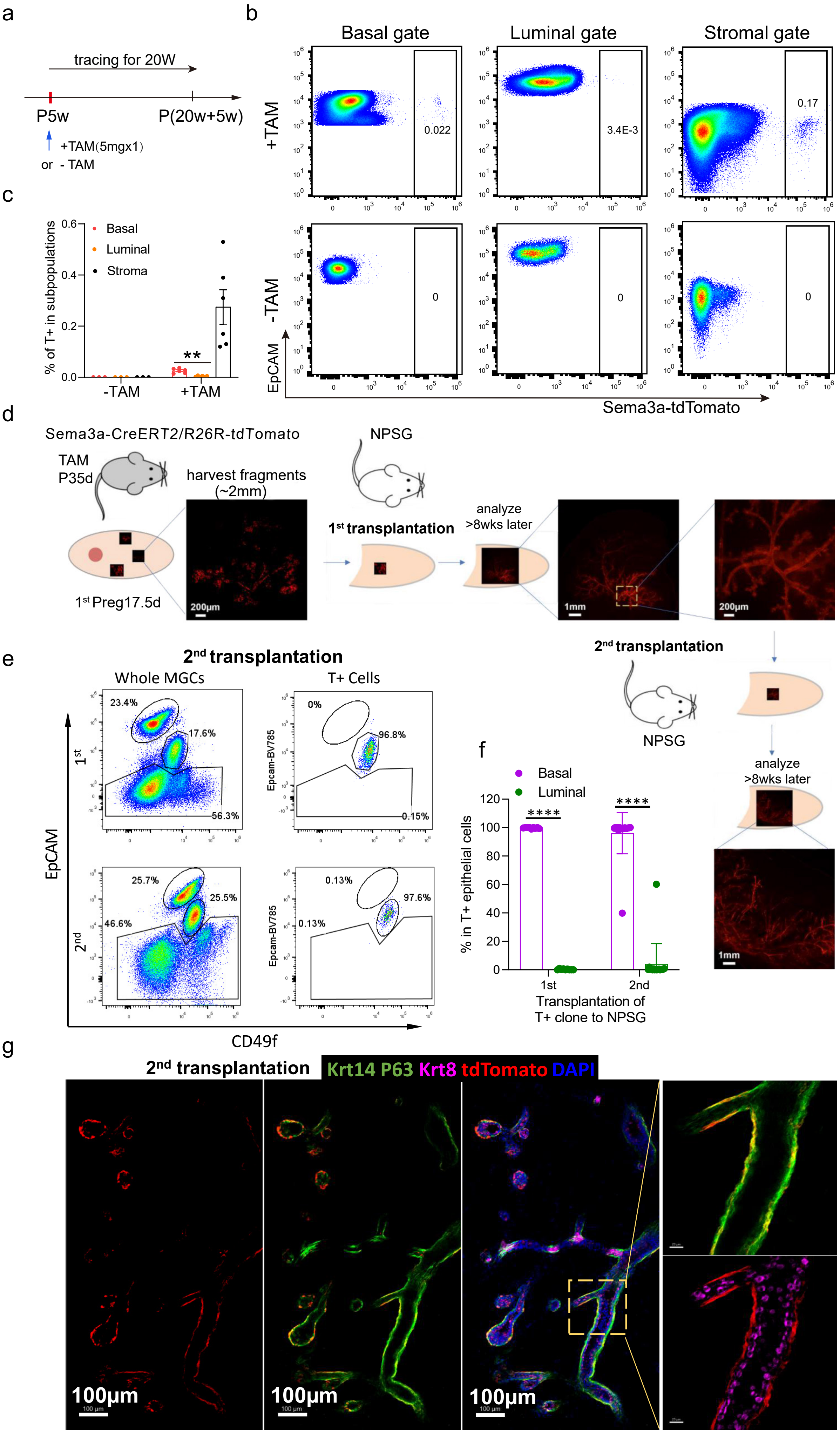

Supplementary Fig. 7 TdTomato-labeled cells in Sema3a-lineage tracing repopulated prosperously as basal population in serial transplantations. (**a**) Schematic diagram showing the tracing strategy of Sema3a$^+$ basal cells from puberty (5-week-old) stage with or without TAM (tamoxifen) treatment for 20 weeks. (**b**) Representative FACS plots showing percentage of tdTomato-labeled cells in basal, luminal, or stromal populations 20 weeks after pubertal tracing. (**c**) Bar chart showing the percentage of tdTomato-labeled cells in different subpopulations. Statistical analysis was performed using two-tailed unpaired t-test. Data were presented as mean ± SEM, n = 3-6. **p<0.01. (**d**) Schematic diagram showing the strategy of serial transplantation of tdTomato-labeled Sema3a$^+$ basal progeny cells. Scale bar, 200μm or 1mm. (**e-f**) Representative FACS plots showing the distribution of tdTomato$^+$ cell in basal and luminal compartments of serial reconstituted mammary glands analyzed by FACS (**e**) and bar chart showing the statistical result of the proportion of basal and luminal cells in tdTomato-labeled epithelial population (**f**). Statistical analysis was performed using two-tailed unpaired t-test. Data were presented as mean ± SEM, n = 9-17. ****p<0.0001. (**g**) Representative images showing tdTomato co-staining with basal markers P63/Krt14, and luminal marker Krt8 in secondary reconstituted mammary glands. Red, tdTomato; green, Krt14/P63; magenta, Krt8; blue, DAPI. Scale bar, 100μm.

Supplementary Fig.8

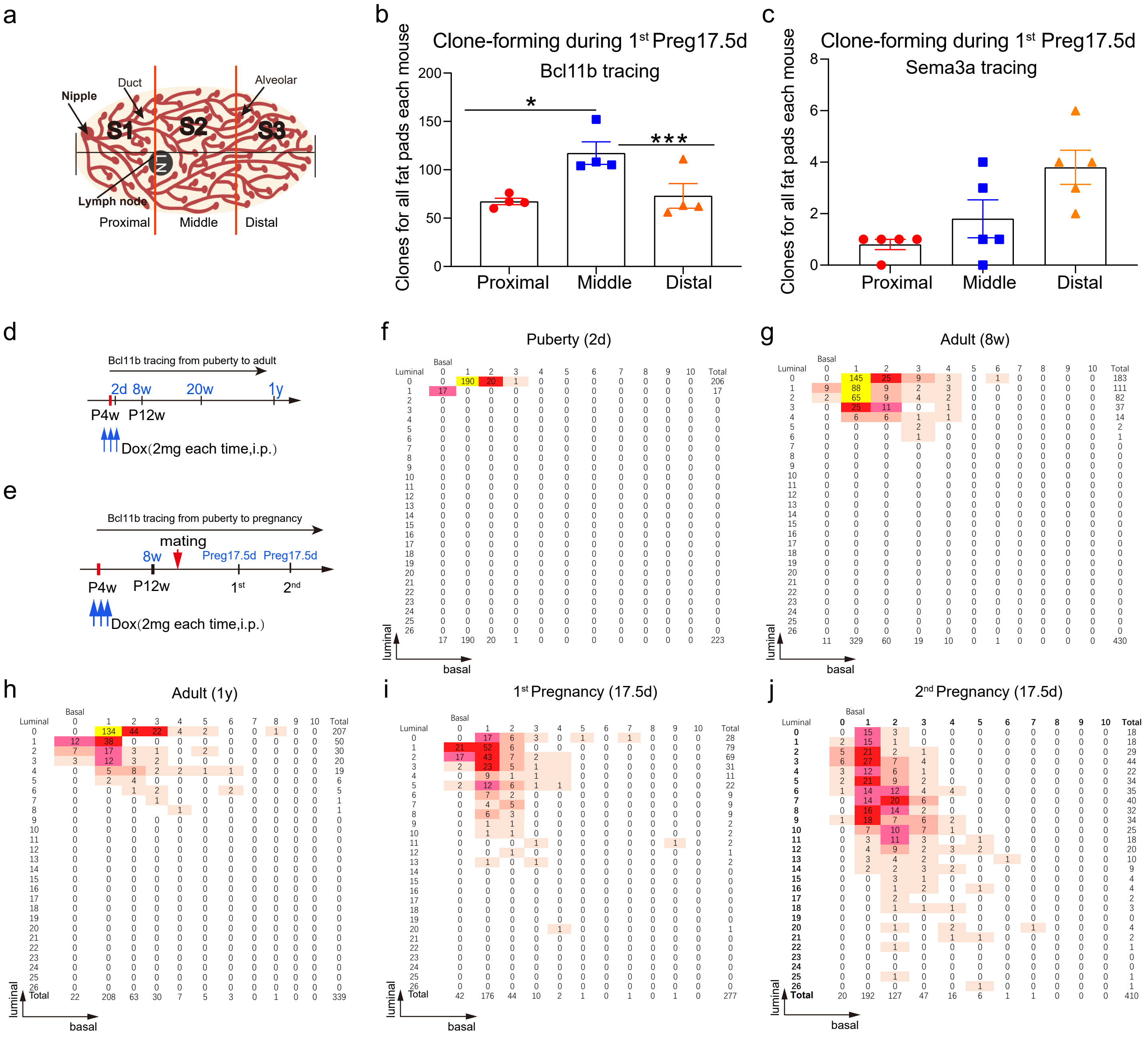

k

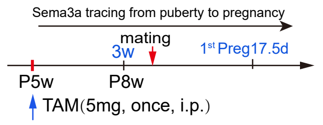

l

1st pregnancy Luminal	Basal 0	<10	10~20	>20	Total
0	0	8	5	22	35
1	1	0	1	2	4
2	0	0	0	2	2
3~20	0	0	0	0	0
>20	1	0	0	0	1
Total	2	8	6	26	42

m

FACS analysis		
Clone	Basal cell	Luminal cell
a	11	0
b	35	0
c	37	0
d	122	0
e	176	0
f	549	0
g	172	1
h	431	1
i	744	1
j	178	2
k	674	3
l	0	68

Supplementary Fig. 8 Distribution and clone analysis of progeny clones of Bcl11b or Sema3a-traced cells at the indicated time points under virgin or multi-pregnancy state after pubertal pulsing. (**a**) Diagram of the strategy for partitioning mammary glands for clone statistical analysis. The mammary gland is considered as an ellipse with its longest axis divided into three equal parts, thus resulting in three regions. The region closest to the nipple is called the proximal region, the region farthest from the nipple is called the distal region, and the region between them is called the middle region. The areas of the three regions are denoted as S1, S2, and S3, respectively. After initially counting the number of clones in each region, the clone counts are normalized based on the different areas of the three regions. (**b**) Bar chart showing clone distribution at different parts with proximal, middle, distal distances from the nipple at 1st Preg17.5 in Bcl11b-lineage tracing from puberty. Statistical analysis was performed using two-tailed unpaired t-test. Data were presented as mean ± SEM, n = 4. *p<0.05, ***p<0.001. (**c**) Bar chart showing clone distribution for Sema3a-lineage tracing in 1st Preg17.5 after pubertal pulsing. Statistical analysis was performed using two-tailed unpaired t-test. Data were presented as mean ± SEM, n = 5. (**d-e**) Schematic diagram showing the lineage-tracing strategy of $Bcl11b^+$ basal cells for induction in pubertal mice at 4 weeks of age, with various chase periods as indicated in virgin mice (2 days, 8 weeks, 1 year) (**d**) or in pregnant mice (1st Preg17.5 days, 2nd Preg17.5 days) (**e**). (**f-j**) Clone analysis (of the composition of each individual clone including the number of basal and luminal cells) by co-immunostaining aSMA /Krt14 with Krt8 and tdTomato of tissue sections for chasing of 2 days (**f**), 8 weeks (**g**), or 1 year (**h**) after pubertal pulsing in virgin mice. And clone analysis for chasing in pregnant mice (**i**,

1st Preg17.5 days; **j**, 2nd Preg17.5 days) after pubertal pulsing in mice. For each group, clones from at least three mice are counted. Specifically, five mice for the 2-day chase in puberty, four mice for the 8-week chase in adulthood, three mice for the 1-year chase in adulthood, three mice for the 1st pregnancy (17.5 days), and three mice for the 2nd pregnancy (17.5 days). (**k**) Schematic diagram showing the lineage-tracing strategy of Sema3a+ basal cells for induction in pubertal mice at 5 weeks of age, followed by continuous chasing until the 1st Preg17.5d. (**l**) Clone analysis by co-immunostaining aSMA /Krt14 with Krt8 and tdTomato of tissue sections shows the clonal expansion of tdTomato+ cells and confirms their predominant distribution in basal cells. Three mice were used for clone analysis. (**m**) FACS analysis indicates that tdTomato+ cells are primary located in the basal layers. TdTomato+ clones from mammary glands of the 1st Preg17.5d mice were dissected, and then basal and luminal cell numbers in all tdToamto+ population of every clone were counted by FACS staining with Lin (CD31/CD45/Ter119), EpCAM and CD49f. And the data comes from clones isolated from three different Sema3a-lineage tracing mice in 1st Preg17.5d after pubertal pulsing.

Supplementary Fig. 9

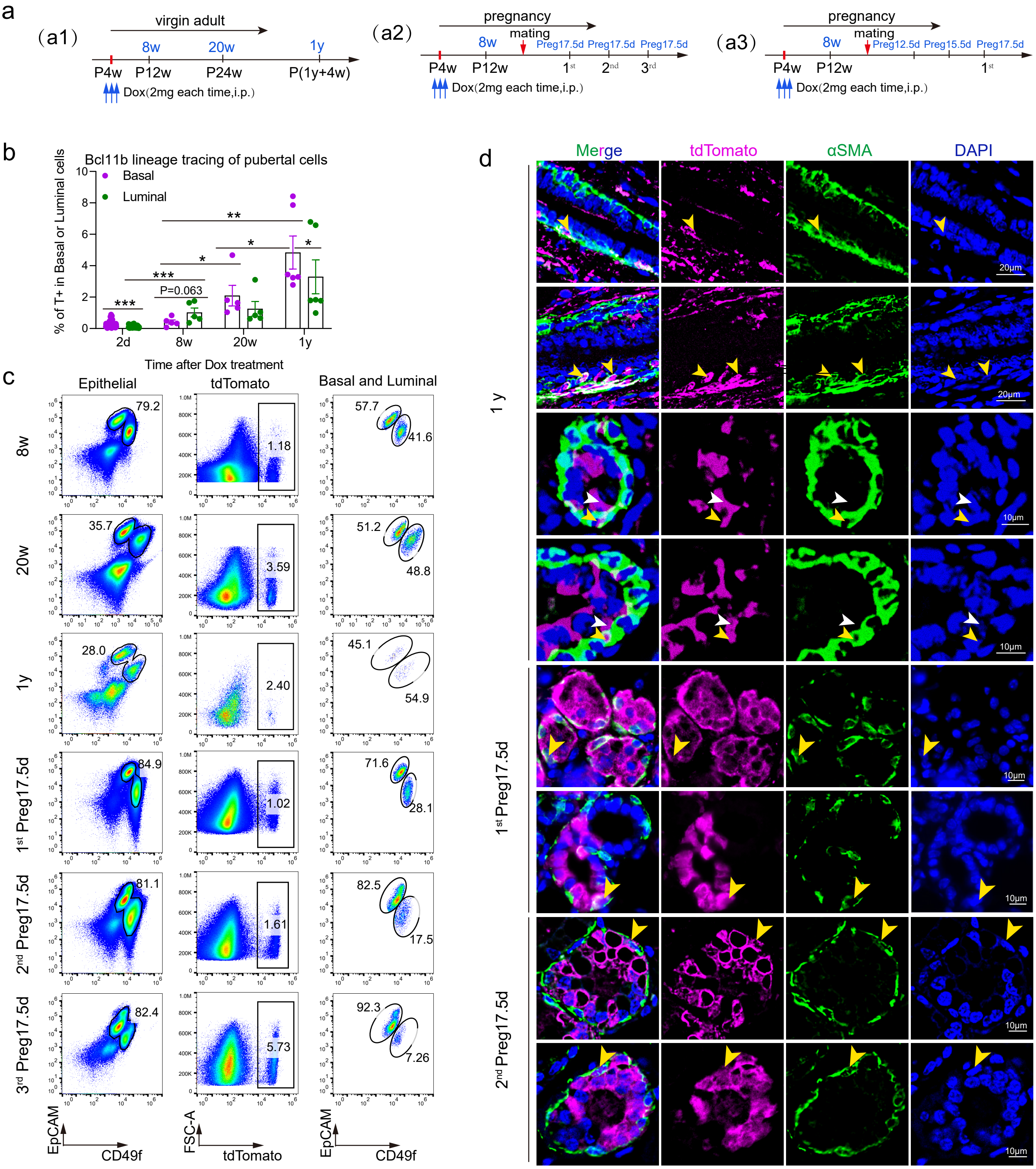

Supplementary Fig. 9 Short and long term Bcl11b-lineage tracing. (**a**) Schematic diagram showing the Bcl11b-lineage tracing strategy for virgin adult chase (a1) and multiple pregnancies chase (a2 and a3) at different time points after pulsing induction at puberty. (**b**) Bar chart showing the percentage of tdTomato-labeled cells in basal or luminal cells at the indicated times after pulsing at puberty. Statistical analysis was performed using two-tailed unpaired t-test. Data were presented as mean ± SEM, n = 5-45. *$p<0.05$, **$p<0.01$, ***$p<0.001$. (**c**) Representative FACS plots showing the composition of Bcl11b traced cells at indicated times after pulsing at puberty. (**d**) Representative images showing clone formation at the indicated times after pulsing at puberty. The yellow and white arrows indicated the tdTomato-labeled basal cells and luminal cells respectively in clones. Green, αSMA; red, tdTomato; blue, DAPI. Scale bar, 10μm or 20μm.

Supplementary Fig.10

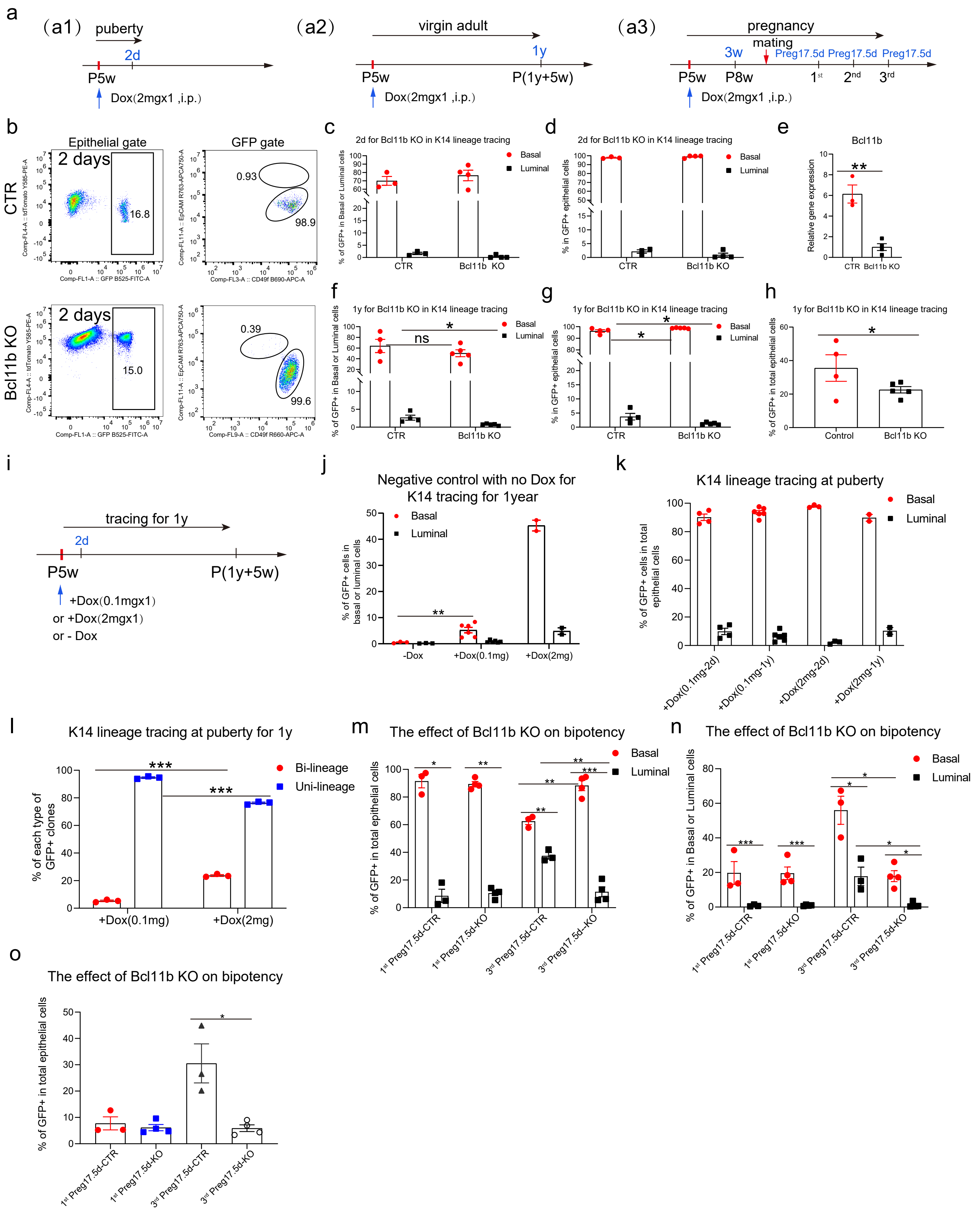

Supplementary Fig. 10 The effect of *Bcl11b* KO in Krt14-lineage tracing. (**a**) Schematic diagram showing the K14-lineage tracing strategy for puberty chase (a1) virgin adult chase (a2) and multiple pregnancies chase (a3) at different time points after pulsing induction at puberty. (**b**) Representative FACS plots showing epithelial and basal cells' labeling efficiency for control and *Bcl11b* KO mice 2 days after Doxycycline induction at puberty. (**c**) Bar chart showing percentage of GFP-labeled cells in basal and luminal populations 2 days after Doxycycline induction at puberty. Data were presented as mean ± SEM, n = 3-4. (**d**) Bar chart showing the composition of total GFP-labeled epithelial cells 2 days after Doxycycline induction at puberty. Data were presented as mean ± SEM, n = 3-4. (**e**) qPCR quantification analysis was performed to detect the expression level of *Bcl11b* in mammary basal cells in control and Bcl11b KO mice relative to that of *β-Actin* 4.5 months after Doxycycline induction at puberty. Data were presented as mean ± SEM, n = 3. $^{**}p<0.01$. (**f**) Bar chart showing percentage of GFP-labeled cells in the basal and luminal populations 1 year after Doxycycline induction at puberty. Data were presented as mean ± SEM, n = 4-5. $^{*}p<0.05$, ns, not significant. (**g**) Bar chart showing the composition of total GFP-labeled epithelial cells 1 year after Doxycycline induction at puberty. Data were presented as mean ± SEM, n = 4-5. $^{*}p<0.05$. (**h**) Bar chart showing percentage of GFP-labeled epithelial cells in total epithelial cells 1 year after Doxycycline induction at puberty. Data were presented as mean ± SEM, n = 4-5. $^{*}p<0.05$. (**i**) Schematic diagram showing the tracing strategy of $K14^{+}$ cells during puberty at 5 weeks old for the indicated chase periods (2 days and 1 year) with no Dox or with a low dose (0.1mg) or high dose (2mg) of Dox treatment. (**j**) Bar chart showing the percentage of GFP-labeled cells in basal or luminal populations 1year after

pubertal K14 tracing. Data were presented as mean ± SEM, n = 2,3-6. **p<0.01. (**k**) Bar chart showing the relative basal and luminal percentage in tdTomato positive mammary epithelial cells for the indicated chase periods (2 days and 1 year) after pubertal tracing with a low dose (0.1mg) or high dose (2mg) of Dox treatment. Data were presented as mean ± SEM, n = 2,3-6. (**l**) Bar chart showing the percentage of GFP^+ bi-lineage and uni-lineage clones formed 1 year after pubertal tracing of $K14^+$ cells with a low dose (0.1mg) or high dose (2mg) of Dox treatment. Data were presented as mean ± SEM, n = 3. ***p<0.001. (**m**) Bar chart showing the composition of GFP-labeled cells at the indicated time points during multiple pregnancies for control and *Bcl11b* KO mice after Doxycycline induction at puberty. Data were presented as mean ± SEM, n = 3-4. *p<0.05, **p<0.01, ***p<0.001. (**n**) Bar chart showing the percentage of GFP-labeled cells in basal and luminal populations at the indicated time points during multiple pregnancies for control and *Bcl11b* KO mice after Doxycycline induction at puberty. Data were presented as mean ± SEM, n = 3-4. *p<0.05, ***p<0.001. (**o**) Bar chart showing percentage of GFP-labeled epithelial cells in total epithelial cells at the indicated time points during multiple pregnancies for control and *Bcl11b* KO mice after Doxycycline induction at puberty. Data were presented as mean ± SEM, n = 3-4. *p<0.05. All above statistical analysis was performed using two-tailed unpaired t-test.
